# Entropy Regularization in the Mean-Field Free-Energy Machine for Combinatorial Optimization

**DOI:** 10.3390/e28101132

**Published:** 2026-10-15

**Authors:** Wei Gao

**Affiliations:** School of Mathematics, Hohai University, Nanjing 211100, China; gaowei@hhu.edu.cn

**Keywords:** free-energy machine, combinatorial optimization, mean-field approximation, entropic regularization, annealing algorithm

## Abstract

The free-energy machine (FEM) relaxes discrete combinatorial optimization by minimizing expected energy plus a temperature-dependent negative-entropy penalty over product distributions. This contribution investigates the computational and mathematical role of that entropy term. At high temperature, entropy drives the optimizer toward the uniform product law; explicit relative-entropy and total-variation bounds quantify this effect, while an influence condition and a restricted Hessian characterize uniqueness, contraction, and the first possible loss of stability. As temperature decreases, the entropy coefficient vanishes. Uniform and variational convergence then connect free energy to the original discrete objective, obtaining an inverse-temperature excess-energy bound, ground-state concentration, and entropy selection among degenerate optima. Extraction bounds, path tracking, perturbation stability, and factorwise complexity further distinguish the effects of entropy, annealing, and replica selection. A batched annealed FEM algorithm is evaluated on exactly enumerable Ising, MaxCut, and Max-3SAT instances; the results reveal the predicted entropy-to-energy transition and show problem-dependent benefits of annealing and replication. For empirical-loss energies, a PAC-Bayes inequality additionally calibrates inverse temperature to sample size. The analysis characterizes entropy-dominated, instability, and energy-dominated regimes without attributing global guarantees to the nonconvex optimizer.

## 1. Introduction

Combinatorial optimization is concerned with selecting a configuration from a finite but typically enormous set so as to minimize a prescribed cost. It underlies graph partitioning, scheduling, routing, satisfiability, coding, resource allocation, and various inference tasks. A convenient generic formulation can be stated by

(1) $$E_{★}=\min_{\sigma \in A}E\left(\sigma \right),\;A=\prod_{i=1}^{d}A_{i},\;\left|A_{i}\right|=K_{i},$$

where *d* is the number of variables, $K_{i}$ is the cardinality of $A_{i}$, and *E* may contain pairwise, higher-order, or global interactions. Even when every variable is binary and *E* is quadratic, this framework includes broad classes of NP-hard problems [1,2,3,4,5]. Exact enumeration scales as $\left|A\right|=\prod_{i}K_{i}$, while worst-case polynomial-time exact algorithms are not expected for unrestricted instances. This gap between a compact algebraic description and an exponentially large configuration space has motivated approximation algorithms, continuous relaxations, local search, tabu and extremal dynamics, and problem-specific heuristics [6,7,8,9].

A specially prominent approach comes from statistical physics. The cost *E* is regarded as an energy and is embedded into the Gibbs distribution

$$\pi_{\beta }\left(\sigma \right)=\frac{e^{-\beta E(\sigma )}}{\sum_{\tau \in A}e^{-\beta E(\tau )}}.$$

At high temperature, corresponding to small inverse temperature β=1/T, the law is diffuse and explores many configurations. At low temperature it concentrates on ground states. Simulated annealing implements this principle with a stochastic Markov chain and has a classical asymptotic convergence theory under logarithmically slow cooling, although the schedules required by the worst-case theory are often impractical [10,11]. The rationale of why low-temperature landscapes contain many competing pure states and metastable basins is delineated by spin-glass theory [12,13,14]. The foregoing ideas further inspired analog, quantum, optical, and digital Ising machines, as well as noisy mean-field solvers and simulated-bifurcation [15,16,17].

Mean-field annealing replaces sampling from the Gibbs law by deterministic optimization over independent marginal distributions. Its early versions inferred self-consistency equations for binary networks and updated their mean magnetizations along a temperature schedule [18,19]. This approach has two superiorities: (1) expectations of sparse local energies can often be evaluated exactly and differentiated without Monte Carlo noise; (2) all marginal probabilities can be updated in parallel. Its limitation is equally appreciable: a product distribution cannot reproduce correlated Gibbs mixtures, and the resulting free-energy landscape need not be convex. Classical variational treatments of graphical models describe this trade-off by means of tractable free-energy approximations [20,21,22]. TAP (Thouless-Anderson-Palmer), Bethe, belief-propagation, and structured variational corrections improve fidelity in selected frameworks but typically sacrifice the simplicity and universality of naive mean field [19,23,24].

The free-energy machine (FEM) of Shen et al. [25] transforms this classical principle into a general GPU-oriented solver. It allows multivalued variables and higher-order interactions, represents each marginal by softmax logits, calculates the expected energy with automatic differentiation or explicit formulas, and runs many independently initialized replicas in a batch. FEM has been demonstrated on MaxCut, balanced graph partitioning, and MaxSAT instances with up to millions of variables. Its central objective for a product law $P_{p}={⨂}_{i=1}^{d}p_{i}$ is denoted by

(2) $$F_{\beta }\left(p\right)=U\left(p\right)-\frac{1}{\beta }H\left(P_{p}\right)=\sum_{\sigma }P_{p}\left(\sigma \right)E\left(\sigma \right)+\frac{1}{\beta }\sum_{\sigma }P_{p}\left(\sigma \right)\log P_{p}\left(\sigma \right),$$

where β>0 is inverse temperature, $U\left(p\right)=E_{P_{p}}E$, and $H\left(P_{p}\right)=\sum_{i}H\left(p_{i}\right)$. In implementation, each marginal is parameterized by logits and a batch of independently initialized product laws is optimized when β increases. The negative-entropy is pivotal to the purposed behavior: at the beginning it favors broad marginals and suppresses premature symmetry breaking; later the expected energy dominates as the temperature 1/β decreases.

Shen et al. [25], moreover, elucidated this annealing process as a qualitative three-stage evolution from an entropy-dominated nearly uniform regime, through a distributional transition, to an energy-dominated low-temperature regime. The aim of this contribution is not to introduce this phenomenological picture, but to determine which parts of it admit explicit finite-temperature bounds, stability criteria, and asymptotic guarantees.

Despite strong empirical results, several basic theoretical problems on (2) remain open at the level of a general finite-state energy. Is the uniform initialization an exact optimum or only an approximation at the starting temperature? When is the high-temperature solution unique, and at what point can distinct branches appear? Does global minimization at low temperature recover a ground state, at what rate, and how does the product constraint behave when the ground state is degenerate? What can be guaranteed after coordinatewise mode rounding or batched sampling? How sensitive is the solution to approximate energies and gradients? Finally, which statements concern the variational objective itself, and which require an optimizer to find its global minimum? Answering these questions is crucial to separate the role of entropy from the separate roles of annealing, numerical optimization, and replica parallelism.

The analysis belongs to a broader field of work on variational free energies. Mean-field accuracy has been studied in terms of nonlinear large deviations, high-temperature conditions, and log-concavity [26,27,28]. The geometry and convergence of coordinate-ascent and gradient-based variational algorithms are analyzed for Ising, community-detection, and Bayesian models [29,30,31,32]. Generalized Gibbs posteriors and their variational approximations connect free-energy minimization to statistical learning [33,34,35,36]. Entropic regularization is employed to control the geometry and expressiveness of variational approximations [37]. The analysis deliberately builds on several classical tools, including mean-field annealing arguments, Dobrushin-type influence bounds, variational convergence, reverse-KL variational representations, and PAC-Bayes change-of-measure inequalities. This work lies in specializing and combining these ingredients for the finite-state product free-energy objective in (2), and in deriving a set of quantitative conclusions that clarify the role of entropy, optimization error, annealing, and replica selection in FEM.

From an information-theoretic perspective, the entropy term in (2) is closely connected to the maximum-entropy principle, which selects a probability law by maximizing entropy subject to available constraints [38]. Such ideas are applied to incorporate prescribed ensemble-averaged observables into statistical-mechanical simulations while minimally perturbing a reference ensemble [39,40]. In the current setting, since

$$-H\left(P_{p}\right)=D_{\mathrm{KL}}\left(P_{p}∥u\right)-\log\left|A\right|,$$

where *u* denotes the uniform law on A, the FEM objective is equivalent, up to an additive constant, to   

$$U\left(p\right)+\frac{1}{\beta }D_{\mathrm{KL}}\left(P_{p}∥u\right).$$

This formulation suggests the more general regularization $U\left(p\right)+\beta^{-1}D_{\mathrm{KL}}\left(P_{p}∥Q\right)$ with a prescribed reference law *Q*, thereby associating the Shannon-entropy term to relative-entropy and cross-entropy representations. If the reference law is updated by virtue of information accumulated during optimization or sampling, the regularization becomes history dependent, conceptually related to adaptive-bias constructions such as metadynamics [41]. In this broader interpretation, 1/β manipulates the trade-off between energy minimization and information-theoretic proximity to the reference law. The analysis in this paper, however, remains restricted to the fixed uniform-reference case in (2).

With the above-mentioned distinction in mind, the FEM-specific contributions are summarized as follows.

1. Starting from the classical reverse-KL representation of mean-field free energy, the FEM objective is elaborated quantitatively in the finite-state product setting. This gets explicit high-temperature bounds on proximity to the uniform law, an influence-based uniqueness and contraction criterion, and a restricted-Hessian condition for the first possible loss of local stability.
2. A FEM-specific low-temperature analysis is obtained, deducing an explicit log|A|/β excess-energy bound, ground-state concentration, entropy selection among degenerate optima, and guarantees for approximate minimizers, mode rounding, sampling, and replicas. Furthermore, robust one-flip optima are showcased to generate low-temperature stationary branches.
3. Canonical annealing and stability ideas are specialized to the FEM dynamics to infer a quantitative path-tracking bound in the contractive regime, a perturbation-stability estimate for approximate energies, and exact factorwise per-step complexity. These results are then incorporated into a batched annealed FEM algorithm and evaluated on exactly enumerable Ising, MaxCut, and Max-3SAT instances.
4. High-dimensional scaling regimes are derived and, for data-dependent energies, a PAC-Bayes oracle inequality is yielded with a statistically calibrated inverse temperature [42,43,44], where these results separate variational guarantees from assumptions about the nonconvex numerical optimizer.

The remainder of the paper is organized as follows. Section 2 develops the variational representation and the high- and low-temperature theory, including the emergence of low-temperature stationary points and all proofs adjacent to their results. Section 3 analyzes annealing, perturbation stability, and computational complexity. A concrete batched algorithm and controlled experiments with exact ground-state certificates are presented in Section 4. The high-dimensional phase diagram and the statistical-learning guarantees are manifested in Section 5. Section 6 specializes the general results to Ising/QUBO (quadratic unconstrained binary optimization), Potts MaxCut (maximum cut), and MaxSAT (maximum satisfiability), and summarizes practical implications. Finally, the limitations and research directions are concluded in Section 7.

## 2. Variational Foundations and Thermal Regimes

This section aims to develop the variational foundations of FEM and characterize its behavior across various temperature regimes. It first determines the Gibbs-projection interpretation and quantifies the mean-field approximation error. The entropy-dominated high-temperature regime is then analyzed in terms of convergence toward the uniform law, uniqueness and contraction under weak interactions, and the onset of local instability. Finally, the energy-dominated low-temperature regime is examined through zero-temperature convergence, solution extraction guarantees, and stationary branches associated with discrete local optima.

Set

$$M=\left|A\right|=\prod_{i=1}^{d}K_{i},\;u\left(\sigma \right)=M^{-1},\;\pi_{\beta }\left(\sigma \right)=Z_{\beta }^{-1}e^{-\beta E(\sigma )},\;Z_{\beta }=\sum_{\sigma \in A}e^{-\beta E(\sigma )}.$$

Let $M=\{{⨂}_{i}p_{i}:p_{i}\in \Delta_{K_{i}}\}$ be the product family and

(3) $$J_{\beta }\left(p\right)=\beta F_{\beta }\left(p\right)=\beta U\left(p\right)-H\left(P_{p}\right).$$

The rescaling removes the singularity of $F_{\beta }$ at β=0 without changing its minimizers for β>0.

**Theorem** **1** (Gibbs projection, existence, and interiority)**.** *For any β>0,*

(4) $$J_{\beta }\left(p\right)=D_{\mathrm{KL}}\left(P_{p}∥\pi_{\beta }\right)-\log Z_{\beta }.$$

*Consequently, FEM is the reverse-KL projection of $\pi_{\beta }$ onto M. The minimum is attained. Every minimizer $p_{\beta }$ is interior: $p_{\beta ,i}\left(a\right)>0$ for each i and $a\in A_{i}$.*

*Define the conditional mean energy*

(5) $$e_{i}\left(a;p_{-i}\right)=E_{P_{p,-i}}\left[E(a,\sigma_{-i})\right].$$

*Then for any global minimizer and any interior stationary point, one gets*

(6) $$p_{i}\left(a\right)=\frac{\exp\{-\beta e_{i}\left(a;p_{-i}\right)\}}{\sum_{b\in A_{i}}\exp\left\{-\beta e_{i}\left(b;p_{-i}\right)\right\}}.$$

*Conversely, every solution of (6) is an interior stationary point of $F_{\beta }$.*

**Proof of** **Theorem 1.** Since $P_{p}$ is a product law,

$$D_{\mathrm{KL}}\left(P_{p}∥\pi_{\beta }\right)=\sum_{\sigma }P_{p}\left(\sigma \right)\left\{\log P_{p}\left(\sigma \right)+\beta E\left(\sigma \right)+\log Z_{\beta }\right\}=J_{\beta }\left(p\right)+\log Z_{\beta },$$

which verifies (4). The product of finite-dimensional closed simplices is compact and $J_{\beta }$ is continuous under the convention 0log0=0; thus, a minimizer exists.

Suppose a minimizer has $p_{i}\left(a\right)=0$. Move mass ε from a positive coordinate *b* to *a*, holding all other marginals fixed. The change in βU is O(ε) since the energy is finite. The new entropy contribution to −H contains εlogε, whose negative magnitude dominates every linear term as ε↓0. The objective therefore decreases, which leads to a contradiction. Hence, each minimizer is interior.

Holding $p_{-i}$ fixed, the terms depending on $p_{i}$ are

$$\sum_{a}p_{i}\left(a\right)e_{i}\left(a;p_{-i}\right)+\beta^{-1}\sum_{a}p_{i}\left(a\right)\log p_{i}\left(a\right).$$

The Lagrange multiplier for $\sum_{a}p_{i}\left(a\right)=1$ gives $e_{i}\left(a\right)+\beta^{-1}\left(\log p_{i}\left(a\right)+1\right)+\lambda_{i}=0$, which is equivalent to (6).

The same deduction also delineates all interior stationary points. Indeed, if *p* is an interior stationary point, then for each *i*, stationarity with respect to variations of $p_{i}$ within the simplex implies that there exists a Lagrange multiplier $\lambda_{i}$ satisfying

$$e_{i}\left(a;p_{-i}\right)+\beta^{-1}\left\{\log p_{i}\left(a\right)+1\right\}+\lambda_{i}=0$$

for each $a\in A_{i}$. Solving these equations together with $\sum_{a}p_{i}\left(a\right)=1$ gives (6). Conversely, if *p* satisfies (6), then

$$e_{i}\left(a;p_{-i}\right)+\beta^{-1}\left\{\log p_{i}\left(a\right)+1\right\}$$

is constant in *a* for each *i*. Hence the directional derivative of $F_{\beta }$ vanishes along every tangent direction of the product simplex, so *p* is an interior stationary point.    □

Theorem 1 clarifies exactly what role entropy plays. It is equivalent, up to the constant $-\beta^{-1}\log M$, to the penalty $\beta^{-1}D_{\mathrm{KL}}\left(P_{p}∥u\right)$. Hence, high temperature pulls the product law toward a uniform prior; it does not penalize statistical dependence, which has already been removed by the feasible set. This interpretation is expressed in terms of the standard information-geometric relation between entropy and relative entropy [45,46].

**Corollary** **1** (Logit gradient)**.** *Let*

$$p_{i}\left(a\right)=\frac{e^{h_{i}\left(a\right)}}{\sum_{b}e^{h_{i}\left(b\right)}}$$

*and*

$$g_{i}\left(a\right)=e_{i}\left(a;p_{-i}\right)+\beta^{-1}\left\{\log p_{i}\left(a\right)+1\right\}.$$

*Then*

(7) $$\frac{\partial F_{\beta }}{\partial h_{i}\left(a\right)}=p_{i}\left(a\right)\left(g_{i}\left(a\right)-\sum_{b}p_{i}\left(b\right)g_{i}\left(b\right)\right),$$

*which is the centered-gradient identity used in FEM. The logits have the gauge invariance $h_{i}\mapsto h_{i}+c_{i}1$.*

**Proof of Corollary** **1.** Holding $p_{-i}$ fixed, the terms of $F_{\beta }$ that depend on $p_{i}$ are

$$\sum_{b}p_{i}\left(b\right)e_{i}\left(b;p_{-i}\right)+\beta^{-1}\sum_{b}p_{i}\left(b\right)\log p_{i}\left(b\right),$$

thus

$$\frac{\partial F_{\beta }}{\partial p_{i}\left(b\right)}=e_{i}\left(b;p_{-i}\right)+\beta^{-1}\left\{\log p_{i}\left(b\right)+1\right\}=g_{i}\left(b\right).$$

Furthermore, the softmax Jacobian satisfies

$$\frac{\partial p_{i}\left(b\right)}{\partial h_{i}\left(a\right)}=p_{i}\left(b\right)\left\{1\left(a=b\right)-p_{i}\left(a\right)\right\}.$$

Therefore, by means of the chain rule,

$$\begin{array}{cc} \frac{\partial F_{\beta }}{\partial h_{i}\left(a\right)} & =\sum_{b}g_{i}\left(b\right)p_{i}\left(b\right)\left\{1\left(a=b\right)-p_{i}\left(a\right)\right\}\\ & =p_{i}\left(a\right)g_{i}\left(a\right)-p_{i}\left(a\right)\sum_{b}p_{i}\left(b\right)g_{i}\left(b\right), \end{array}$$

which is (7).    □

### 2.1. Mean-Field Approximation Error to the Gibbs Law

Define the reverse-KL approximation error by

(8) $$A_{\beta }=\min_{P\in M}D_{\mathrm{KL}}\left(P∥\pi_{\beta }\right).$$

The exact Gibbs free energy is $-\beta^{-1}\log Z_{\beta }$, whereas the optimal mean-field free energy is

(9) $$F_{\beta }^{\mathrm{MF}}=-\beta^{-1}\log Z_{\beta }+\beta^{-1}A_{\beta }.$$

**Proposition** **1** (Two universal projection bounds)**.** *Let $R_{E}=\mathrm{osc}\left(E\right)=\max_{\sigma }E\left(\sigma \right)-\min_{\sigma }E\left(\sigma \right)$. Then*

(10) $$0\leq A_{\beta }\leq \min\left\{\frac{\beta^{2}R_{E}^{2}}{8},\;\log M\right\}.$$

*Specifically, if $P_{p_{\beta }}$ is a global FEM minimizer (Throughout this paper, statements about a “global FEM minimizer” concern the mathematical minimum of (2)), then*

(11) $$d_{\mathrm{TV}}\left(P_{p_{\beta }},\pi_{\beta }\right)\leq \sqrt{A_{\beta }/2}\leq \frac{\beta R_{E}}{4}$$

*whenever the high-temperature bound is used.*

**Proof of Proposition** **1.** Employing the uniform product law as a competitor,

$$A_{\beta }\leq D_{\mathrm{KL}}\left(u∥\pi_{\beta }\right)=\log E_{u}\exp\left\{-\beta \left(E-E_{u}E\right)\right\}\leq \beta^{2}R_{E}^{2}/8,$$

where the last step follows from Hoeffding’s lemma. If $\sigma_{★}$ is a ground state, the point mass $\delta_{\sigma_{★}}$ is also a product law and

$$A_{\beta }\leq D_{\mathrm{KL}}\left(\delta_{\sigma_{★}}∥\pi_{\beta }\right)=\log\sum_{\sigma }e^{-\beta (E\left(\sigma \right)-E_{★})}\leq \log M.$$

Therefore, Equation (11) is obtained in light of Pinsker’s inequality and the minimizing identity $D_{\mathrm{KL}}\left(P_{p_{\beta }}∥\pi_{\beta }\right)=A_{\beta }$.

If $p_{\beta }$ is a global FEM minimizer, then Theorem 1 and the definition of $A_{\beta }$ imply $D_{\mathrm{KL}}\left(P_{p_{\beta }}∥\pi_{\beta }\right)=A_{\beta }$. Applying Pinsker’s inequality therefore obtains

$$d_{\mathrm{TV}}\left(P_{p_{\beta }},\pi_{\beta }\right)\leq \sqrt{\frac{1}{2}D_{\mathrm{KL}}\left(P_{p_{\beta }}∥\pi_{\beta }\right)}=\sqrt{\frac{A_{\beta }}{2}}.$$

In light of the high-temperature bound $A_{\beta }\leq \beta^{2}R_{E}^{2}/8$ from (10), one further gets

$$d_{\mathrm{TV}}\left(P_{p_{\beta }},\pi_{\beta }\right)\leq \sqrt{\frac{A_{\beta }}{2}}\leq \sqrt{\frac{\beta^{2}R_{E}^{2}}{16}}=\frac{\beta R_{E}}{4},$$

which confirms (11).    □

The first bound in (10) is useful only in a genuine high-temperature scaling. The second is temperature-uniform but does not say that the mean-field law reproduces correlations of $\pi_{\beta }$. Reverse KL is mode-seeking, especially when the Gibbs law is multimodal.

### 2.2. The Entropy-Dominated Regime

#### 2.2.1. Uniform Limit and Its First Correction

Let $p_{i}^{u}\left(a\right)=K_{i}^{-1}$ and $P_{u}={⨂}_{i}p_{i}^{u}=u$.

**Theorem** **2** (Quantitative uniform regime)**.** *For any global minimizer $p_{\beta }$,*

(12) $$\begin{array}{cc} D_{\mathrm{KL}}\left(P_{p_{\beta }}∥u\right) & =\log M-H\left(P_{p_{\beta }}\right)\leq \beta R_{E}, \end{array}$$

(13) $$\begin{array}{cc} d_{\mathrm{TV}}\left(P_{p_{\beta }},u\right) & \leq \sqrt{\beta R_{E}/2}, \end{array}$$

(14) $$\begin{array}{cc} \frac{1}{d}\sum_{i=1}^{d}d_{\mathrm{TV}}\left(p_{\beta ,i},p_{i}^{u}\right)\; & \leq \sqrt{\frac{\beta R_{E}}{2d}}. \end{array}$$

*Hence, $p_{\beta }\to p^{u}$ as β↓0. Moreover, the locally unique solution emanating from $p^{u}$ has expansion*

(15) $$p_{\beta ,i}\left(a\right)=\frac{1}{K_{i}}-\frac{\beta }{K_{i}}\left(e_{i}\left(a;p_{-i}^{u}\right)-U\left(p^{u}\right)\right)+O\left(\beta^{2}\right).$$

*Therefore, exact uniformity at positive β requires all conditional mean energies $e_{i}\left(a;p_{-i}^{u}\right)$ to be constant in a.*

**Proof of Theorem** **2.** Since $p_{\beta }$ is a global minimizer of $J_{\beta }\left(p\right)=\beta U\left(p\right)-H\left(P_{p}\right)$, its objective value cannot exceed that of the feasible uniform product law $p^{u}$. Hence $J_{\beta }\left(p_{\beta }\right)\leq J_{\beta }\left(p^{u}\right)$, or equivalently,   

$$\beta U\left(p_{\beta }\right)-H\left(P_{p_{\beta }}\right)\leq \beta U\left(p^{u}\right)-H\left(P_{p^{u}}\right).$$

Since $P_{p^{u}}=u$ is uniform on the *M*-element configuration space A, $H(P_{p^{u}})=\log M$. Therefore,

$$\beta U\left(p_{\beta }\right)-H\left(P_{p_{\beta }}\right)\leq \beta U\left(p^{u}\right)-\log M.$$

Thus,

$$D_{\mathrm{KL}}\left(P_{p_{\beta }}∥u\right)=\log M-H\left(P_{p_{\beta }}\right)\leq \beta \left\{U\left(p^{u}\right)-U\left(p_{\beta }\right)\right\}\leq \beta R_{E},$$

and (13) is determined by Pinsker’s inequality (apply it to $P_{p_{\beta }}$ and *u*) and (12). Product additivity of KL and Cauchy–Schwarz infer

$$\frac{1}{d}\sum_{i}d_{\mathrm{TV}}\left(p_{\beta ,i},u_{i}\right)\leq \frac{1}{d}\sum_{i}\sqrt{D_{\mathrm{KL}}\left(p_{\beta ,i}∥u_{i}\right)/2}\leq \sqrt{D_{\mathrm{KL}}\left(P_{p_{\beta }}∥u\right)/\left(2d\right)}.$$

The mean-field map is the constant map $p^{u}$ at β=0, so a unique smooth branch locally is obtained by the implicit function theorem (IFT). Differentiating the softmax equation at zero determines

$${\left.\frac{d}{d\beta }p_{\beta ,i}\left(a\right)\right|}_{\beta =0}=-K_{i}^{-1}\left(e_{i}\left(a;p_{-i}^{u}\right)-K_{i}^{-1}\sum_{b}e_{i}\left(b;p_{-i}^{u}\right)\right).$$

Since $p_{i}^{u}\left(b\right)=1/K_{i}$ and $P_{p^{u}}=p_{i}^{u}⊗P_{p^{u},-i}$, the average conditional energy satisfies

$$\frac{1}{K_{i}}\sum_{b\in A_{i}}e_{i}\left(b;p_{-i}^{u}\right)=\sum_{b\in A_{i}}p_{i}^{u}\left(b\right)E_{P_{p^{u},-i}}\left[E\left(b,\sigma_{-i}\right)\right]=E_{P_{p^{u}}}\left[E\left(\sigma \right)\right]=U\left(p^{u}\right).$$

Hence,

$${\left.\frac{d}{d\beta }p_{\beta ,i}\left(a\right)\right|}_{\beta =0}=-\frac{1}{K_{i}}\left[e_{i}\left(a;p_{-i}^{u}\right)-U\left(p^{u}\right)\right].$$

Using $p_{0,i}\left(a\right)=1/K_{i}$, the first-order Taylor expansion of the smooth branch $p_{\beta }$ at β=0 yields (15).    □

Equation (15) formalizes the statement that uniform initialization maximizes entropy. The optimizer starts moving immediately unless symmetry cancels the first-order energy signal.

#### 2.2.2. Weak Interactions: Uniqueness and Global Contraction

For i≠j, denote the influence of coordinate *j* on the energy preference at coordinate *i* as

(16) $$\begin{array}{cc} c_{ij}=\max_{a,b\in A_{i}}\;\max_{x_{-i-j}}\;\max_{s,t\in A_{j}}| & E\left(a,s,x_{-i-j}\right)-E\left(b,s,x_{-i-j}\right)\\ \; & -E\left(a,t,x_{-i-j}\right)+E\left(b,t,x_{-i-j}\right)|. \end{array}$$

Set $C=\max_{i}\sum_{j\neq i}c_{ij}$. Let $S_{\beta }$ be the parallel softmax map on the right-hand side of (6), and equip the product of marginal simplices with

$$d_{\infty }\left(p,q\right)=\max_{i}d_{\mathrm{TV}}\left(p_{i},q_{i}\right).$$

**Theorem** **3** (High-temperature uniqueness)**.** *For all p,q,*

(17) $$d_{\infty }\left(S_{\beta }p,S_{\beta }q\right)\leq \alpha_{\beta }d_{\infty }\left(p,q\right),\;\alpha_{\beta }=\frac{\beta C}{4}.$$

*If βC<4, the FEM stationary point is unique and is the unique global minimizer. Parallel mean-field iteration converges geometrically:*

(18) $$d_{\infty }\left(p^{\left(t\right)},p_{\beta }\right)\leq \alpha_{\beta }^{t}d_{\infty }\left(p^{\left(0\right)},p_{\beta }\right).$$

*The damped update $p^{\left(t+1\right)}=\left(1-\eta \right)p^{\left(t\right)}+\eta S_{\beta }p^{\left(t\right)}$, 0<η≤1, has contraction factor $1-\eta (1-\alpha_{\beta })$.*

**Lemma** **1** (Softmax comparison)**.** *For finite vectors f,g and $s_{\beta }\left(f\right)\left(a\right)\propto e^{-\beta f(a)}$,*

(19) $$d_{\mathrm{TV}}\left(s_{\beta }\left(f\right),s_{\beta }\left(g\right)\right)\leq \tanh\;\left(\frac{\beta \mathrm{osc}(f-g)}{4}\right)\leq \frac{\beta \mathrm{osc}(f-g)}{4}.$$

**Proof.**  Let $P=s_{\beta }\left(f\right)$, $Q=s_{\beta }\left(g\right)$, and R(a)=P(a)/Q(a). Then

$$\log R\left(a\right)=-\beta \left(f\left(a\right)-g\left(a\right)\right)+\log\left(Z_{g}/Z_{f}\right),$$

thus

osc(logR)=βosc(f−g)=:L.

Let $m=\min_{a}R\left(a\right)$ and $M=\max_{a}R\left(a\right)$. Then $M/m\leq e^{L}$, while

$$E_{Q}R=\sum_{a}Q\left(a\right)R\left(a\right)=1.$$

Moreover,

$$d_{\mathrm{TV}}\left(P,Q\right)=\frac{1}{2}E_{Q}\left|R-1\right|.$$

Since R∈[m,M] and $E_{Q}R=1$, convexity of r↦|r−1| verifies

$$d_{\mathrm{TV}}\left(P,Q\right)\leq \frac{\left(M-1)(1-m\right)}{M-m}.$$

Writing $M/m=t^{2}$, the bound above is maximized over m≤1≤M at $m=t^{-1}$ and M=t, yielding

$$d_{\mathrm{TV}}\left(P,Q\right)\leq \frac{t-1}{t+1}.$$

Using $t\leq e^{L/2}$, one gives

$$d_{\mathrm{TV}}\left(P,Q\right)\leq \frac{e^{L/2}-1}{e^{L/2}+1}=\tanh\left(L/4\right).$$

Substituting L=βosc(f−g) yields the first inequality in (19), and the second in terms of tanhx≤x for x≥0.    □

**Proof of Theorem** **3.** For given $a,b\in A_{i}$, telescope from $P_{p,-i}$ to $P_{q,-i}$ one marginal at a time. The definition of $c_{ij}$ and the variational characterization of total variation infer

$$\left|\left[e_{i}\left(a;p_{-i}\right)-e_{i}\left(b;p_{-i}\right)\right]-\left[e_{i}\left(a;q_{-i}\right)-e_{i}\left(b;q_{-i}\right)\right]\right|\leq \sum_{j\neq i}c_{ij}d_{\mathrm{TV}}\left(p_{j},q_{j}\right).$$

Taking the maximum over a,b bounds the oscillation of the two conditional energy vectors. In light of Lemma 1, one determines

$$d_{\mathrm{TV}}\left({\left(S_{\beta }p\right)}_{i},{\left(S_{\beta }q\right)}_{i}\right)\leq \frac{\beta }{4}\sum_{j\neq i}c_{ij}d_{\mathrm{TV}}\left(p_{j},q_{j}\right),$$

which proves (17).

If βC<4, then $\alpha_{\beta }=\beta C/4<1$, so (17) reveals that $S_{\beta }$ is a contraction on the complete metric space $\left(\prod_{i}\Delta_{K_{i}},d_{\infty }\right)$. Banach’s fixed-point theorem hence guarantees a unique fixed point $p_{\beta }$. Moreover, for the parallel iteration $p^{\left(t+1\right)}=S_{\beta }p^{\left(t\right)}$,

$$d_{\infty }\left(p^{\left(t+1\right)},p_{\beta }\right)=d_{\infty }\left(S_{\beta }p^{\left(t\right)},S_{\beta }p_{\beta }\right)\leq \alpha_{\beta }d_{\infty }\left(p^{\left(t\right)},p_{\beta }\right),$$

and iteration of this inequality determines

$$d_{\infty }\left(p^{\left(t\right)},p_{\beta }\right)\leq \alpha_{\beta }^{t}d_{\infty }\left(p^{\left(0\right)},p_{\beta }\right),$$

which proves (18).

By means of Theorem 1, a global minimizer $p^{★}$ of $F_{\beta }$ exists and is interior. Every such minimizer meets the softmax fixed-point Equation (6), and thus $S_{\beta }p^{★}=p^{★}$. Since $S_{\beta }$ has a unique fixed point, $p^{★}=p_{\beta }$; therefore $p_{\beta }$ is the unique global minimizer.

Finally, define the damped map

$$T_{\beta ,\eta }\left(p\right)=\left(1-\eta \right)p+\eta S_{\beta }p.$$

According to convexity of total variation under mixtures, for each *i*,

$$\begin{array}{cc} d_{\mathrm{TV}}\left({\left(T_{\beta ,\eta }p\right)}_{i},{\left(T_{\beta ,\eta }q\right)}_{i}\right) & \leq \left(1-\eta \right)d_{\mathrm{TV}}\left(p_{i},q_{i}\right)+\eta d_{\mathrm{TV}}\left({\left(S_{\beta }p\right)}_{i},{\left(S_{\beta }q\right)}_{i}\right)\\ & \leq \left[\left(1-\eta \right)+\eta \alpha_{\beta }\right]d_{\infty }\left(p,q\right). \end{array}$$

Taking the maximum over *i* gets

$$d_{\infty }\left(T_{\beta ,\eta }p,T_{\beta ,\eta }q\right)\leq \left[1-\eta (1-\alpha_{\beta })\right]d_{\infty }\left(p,q\right),$$

thus the damped update has contraction factor $1-\eta (1-\alpha_{\beta })$.    □

The condition is sufficient, not necessary. It is a general discrete-state analogue of the familiar high-temperature Dobrushin condition. Its failure marks the end of a theorem-certified single-basin regime, not necessarily the physical transition itself; see [47] for the corresponding Gibbs measure theory.

#### 2.2.3. Hessian Criterion and the First Bifurcation

Let the tangent space of the product simplex be

$$T=\left\{v=\left(v_{1},\ldots ,v_{d}\right):\sum_{a\in A_{i}}v_{i}\left(a\right)=0\;\mathrm{for}\;\mathrm{every}\;i\right\}.$$

At an interior point *p*, the restricted Hessian of $J_{\beta }$ is formalized by

(20) $$H_{\beta }\left(p\right)\left[v,v\right]=\sum_{i,a}\frac{v_{i}{\left(a\right)}^{2}}{p_{i}\left(a\right)}+\beta \;\nabla^{2}U\left(p\right)\left[v,v\right],\;v\in T.$$

**Proposition** **2** (Local stability threshold)**.** *Let p be an interior stationary point of $J_{\beta }$, and let T be the tangent space defined above. Define*

$$D\left(p\right):=\mathrm{diag}\left\{1/p_{i}\left(a\right)\right\},\;H_{\beta }\left(p\right):=D\left(p\right)+\beta \nabla^{2}U\left(p\right).$$

*Then, for any v∈T,*

$$v^{⊤}H_{\beta }\left(p\right)v=\sum_{i,a}\frac{v_{i}{\left(a\right)}^{2}}{p_{i}\left(a\right)}+\beta \nabla^{2}U\left(p\right)\left[v,v\right].$$

*If $H_{\beta }\left(p\right)$ is positive definite on T, i.e.,*

$$v^{⊤}H_{\beta }\left(p\right)v>0\;\mathrm{for}\;\mathrm{every}\;v\in T\setminus \left\{0\right\},$$

*then p is a strict local minimum. If there exists v∈T∖{0} such that*

$$v^{⊤}H_{\beta }\left(p\right)v<0,$$

*then p has a feasible direction of negative curvature and is locally unstable.*

*Along a smooth stationary branch p=p(β), a necessary condition for loss of local stability is that the smallest eigenvalue of the restricted Hessian reaches zero. Equivalently, after the change of variables $w=D{\left(p\right)}^{1/2}v$, the feasible tangent space becomes $D{\left(p\right)}^{1/2}T$, and the normalized Hessian is $D{\left(p\right)}^{-1/2}H_{\beta }\left(p\right)D{\left(p\right)}^{-1/2}$. Thus the condition can be written as*

(21) $$\lambda_{\min}\left(D{\left(p\right)}^{-1/2}H_{\beta }\left(p\right)D{\left(p\right)}^{-1/2}{|}_{D{\left(p\right)}^{1/2}T}\right)=0.$$

*This condition provides an instance-specific candidate critical inverse temperature $\beta_{c}$ for the first possible loss of local stability along the branch.*

**Proof of Proposition** **2.** Since $J_{\beta }\left(p\right)=\beta U\left(p\right)+\sum_{i,a}p_{i}\left(a\right)\log p_{i}\left(a\right)$ and $\frac{d^{2}}{dx^{2}}\left(x\log x\right)=\frac{1}{x}$, the Hessian of $J_{\beta }$ at an interior point *p* is

$$H_{\beta }\left(p\right)=D\left(p\right)+\beta \nabla^{2}U\left(p\right),\;D\left(p\right)=\mathrm{diag}\left\{1/p_{i}\left(a\right)\right\}.$$

Feasible perturbations must preserve the normalization of every marginal, so they satisfy $\sum_{a\in A_{i}}v_{i}\left(a\right)=0$ for each *i* and hence belong to T. Therefore, for v∈T,

$$v^{⊤}H_{\beta }\left(p\right)v=\sum_{i,a}\frac{v_{i}{\left(a\right)}^{2}}{p_{i}\left(a\right)}+\beta \nabla^{2}U\left(p\right)\left[v,v\right],$$

which derives (20).

Assume *p* is an interior stationary point. For each feasible direction v∈T, the first directional derivative vanishes. Thus,

$$J_{\beta }\left(p+\varepsilon v\right)-J_{\beta }\left(p\right)=\frac{\varepsilon^{2}}{2}v^{⊤}H_{\beta }\left(p\right)v+o\left(\varepsilon^{2}\right).$$

If $H_{\beta }\left(p\right)$ is positive definite on T, the right-hand side is positive for every sufficiently small nonzero feasible perturbation, hence *p* is a strict local minimum. Conversely, if there exists v∈T∖{0} with $v^{⊤}H_{\beta }\left(p\right)v<0$, then sufficiently small perturbations in the direction *v* decrease the objective, so the stationary point is locally unstable.

Finally, consider a smooth stationary branch p(β). Since p(β) is interior, D(p(β)) is positive definite. Under the invertible change of variables

$$w=D{\left(p\right)}^{1/2}v,$$

a feasible direction v∈T corresponds exactly to $w\in D{\left(p\right)}^{1/2}T$, and

$$v^{⊤}H_{\beta }\left(p\right)v=w^{⊤}D{\left(p\right)}^{-1/2}H_{\beta }\left(p\right)D{\left(p\right)}^{-1/2}w.$$

Hence the normalized Hessian restricted to $D{\left(p\right)}^{1/2}T$ has the same inertia as the original Hessian restricted to T. Along a smooth branch the restricted Hessian varies continuously with β, so its eigenvalues vary continuously as well. Therefore, a change from positive-definite to indefinite curvature can occur only when the smallest restricted eigenvalue first reaches zero. Consequently, a necessary condition for loss of local stability is

$$\lambda_{\min}\left(D{\left(p\right)}^{-1/2}H_{\beta }\left(p\right)D{\left(p\right)}^{-1/2}{|}_{D{\left(p\right)}^{1/2}T}\right)=0,$$

which gives (21).    □

The uniform law itself need not remain a stationary point for β>0. Accordingly, one should monitor the Hessian along the annealed branch rather than assert a universal phase transition at a fixed temperature.

#### 2.2.4. Monotonicity Along Global Optima

**Proposition** **3** (Energy-entropy monotonicity)**.** *Let $T_{1}>T_{2}>0$ and let $p^{\left(k\right)}$ globally minimize $F_{T_{k}}=U-T_{k}H$. Then*

(22) $$H\left(P_{p^{\left(1\right)}}\right)\geq H\left(P_{p^{\left(2\right)}}\right),\;U\left(p^{\left(1\right)}\right)\geq U\left(p^{\left(2\right)}\right).$$

*Thus, along globally optimized annealing, decreasing temperature cannot increase the expected energy or increase entropy. This conclusion may fail for a numerical trajectory that switches between non-global basins.*

**Proof of Proposition** **3.** Optimality at $T_{1}$ and $T_{2}$ gives

$$U_{1}-T_{1}H_{1}\leq U_{2}-T_{1}H_{2},\;U_{2}-T_{2}H_{2}\leq U_{1}-T_{2}H_{1}.$$

Adding the inequalities yields $\left(T_{1}-T_{2}\right)\left(H_{1}-H_{2}\right)\geq 0$, hence $H_{1}\geq H_{2}$. Substitution into either inequality determines $U_{1}\geq U_{2}$.    □

### 2.3. The Energy-Dominated Regime

Let

$$G=\left\{\sigma :E\left(\sigma \right)=E_{★}\right\},\;\Delta =\min_{\sigma \notin G}\left\{E\left(\sigma \right)-E_{★}\right\},$$

with the concentration statement below interpreted as vacuous if G=A.

**Theorem** **4** (Zero-temperature limit, rate, and entropy selection)**.** *Let $p_{T}$ be any global minimizer of $F_{T}=U-TH$. Then*

(23) $$0\leq U\left(p_{T}\right)-E_{★}\leq T\log M=\frac{\log M}{\beta }.$$

*The functionals $F_{T}$ converge uniformly, and therefore* Γ*-converge, to U on the compact product simplex as T↓0. Every accumulation point $p_{0}$ meets*

(24) $$P_{p_{0}}\left(G\right)=1,\;H\left(P_{p_{0}}\right)=\max\left\{H\left(P_{p}\right):P_{p}\left(G\right)=1\right\}.$$

*In addition,*

(25) $$P_{p_{T}}\left(G^{c}\right)\leq \frac{T\log M}{\Delta }.$$

*If the ground state is unique, $P_{p_{T}}$ converges in total variation to its point mass.*

The application of Γ-convergence follows the standard variational framework in [48]; in the current finite-dimensional setting, the stronger uniform convergence makes the argument particularly direct.

**Proof of Theorem** **4.** Compare $p_{T}$ with a ground-state point mass. Since point masses have zero entropy, one gets

$$U\left(p_{T}\right)-TH\left(P_{p_{T}}\right)\leq E_{★}.$$

The Equation (23) is obtained by virtue of $U\left(p_{T}\right)\geq E_{★}$ and $H(P_{p_{T}})\leq \log M$. It is noteworthy that $\sup_{p}\left|F_{T}\left(p\right)-U\left(p\right)\right|\leq T\log M$, so convergence is uniform. Moreover, since $0\leq H(P_{p})\leq \log M$,

$$\underset{p\in M}{\sup}\left|F_{T}\left(p\right)-U\left(p\right)\right|\leq T\log M\to 0,$$

thus $F_{T}$ converges uniformly to *U* on the compact product simplex M. Specifically, for any $T_{n}↓0$ and $p_{n}\to p$, continuity of *U* determines

$$F_{T_{n}}\left(p_{n}\right)=U\left(p_{n}\right)+o\left(1\right)\to U\left(p\right),$$

which implies the Γ-liminf inequality; the constant recovery sequence $p_{n}=p$ gives the corresponding Γ-limsup inequality. Hence, $F_{T}$ Γ-converges to *U*. Furthermore, compactness implies that any sequence of minimizers $p_{T_{n}}$ admits a convergent subsequence, say $p_{T_{n}}\to p_{0}$. For any q∈M, optimality obtains $F_{T_{n}}\left(p_{T_{n}}\right)\leq F_{T_{n}}\left(q\right)$. Passing to the limit using uniform convergence and continuity of *U* yields $U\left(p_{0}\right)\leq U\left(q\right)$. Since *q* is arbitrary, $p_{0}$ is a global minimizer of *U*.

For any product law $P_{p}$, $U\left(p\right)=E_{★}$ if and only if it assigns probability one to G. To infer entropy selection, compare $p_{T}$ with any *q* satisfying $U\left(q\right)=E_{★}$:

$$U\left(p_{T}\right)-E_{★}\leq T\left\{H\left(P_{p_{T}}\right)-H\left(P_{q}\right)\right\}.$$

Since $U\left(p_{T}\right)\geq E^{★}$ and T>0, the preceding inequality implies $H\left(P_{p_{T}}\right)\geq H\left(P_{q}\right)$ for each *q* meeting $U\left(q\right)=E^{★}$. Let $T_{n}↓0$ be a subsequence such that $p_{T_{n}}\to p_{0}$. As shown above, $p_{0}$ minimizes *U*, and hence $U\left(p_{0}\right)=E^{★}$, or equivalently $P_{p_{0}}\left(G\right)=1$. Since the state space is finite and x↦−xlogx is continuous on [0,1] under the convention 0log0=0, entropy is continuous. Therefore,

$$H\left(P_{p_{T_{n}}}\right)⟶H\left(P_{p_{0}}\right).$$

Passing to the limit in $H\left(P_{p_{T_{n}}}\right)\geq H\left(P_{q}\right)$ gets $H\left(P_{p_{0}}\right)\geq H\left(P_{q}\right)$ for each *q* with $U\left(q\right)=E^{★}$. Since $U\left(q\right)=E^{★}$ if and only if $P_{q}\left(G\right)=1$, and $p_{0}$ itself satisfies this condition,

$$H\left(P_{p_{0}}\right)=\max\left\{H\left(P_{p}\right):P_{p}\left(G\right)=1\right\},$$

which, together with $P_{p_{0}}\left(G\right)=1$, proves (24).

Finally, since $E\left(\sigma \right)-E^{★}=0$ for σ∈G and $E\left(\sigma \right)-E^{★}\geq \Delta$ for $\sigma \in G^{c}$,

$$\begin{array}{cc} U\left(p_{T}\right)-E^{★} & =\sum_{\sigma \in G^{c}}P_{p_{T}}\left(\sigma \right)\left(E\left(\sigma \right)-E^{★}\right)\\ & \geq \Delta \sum_{\sigma \in G^{c}}P_{p_{T}}\left(\sigma \right)=\Delta P_{p_{T}}\left(G^{c}\right). \end{array}$$

Combining this lower bound with (23) gives

$$\Delta P_{p_{T}}\left(G^{c}\right)\leq U\left(p_{T}\right)-E^{★}\leq T\log M,$$

and hence yields (25).    □

Since product support of $P_{p}$ is a Cartesian set $\prod_{i}\mathrm{supp}\left(p_{i}\right)$, Equation (24) has a concrete combinatorial interpretation.

**Corollary** **2** (Rectangular selection under degeneracy)**.** *Let*

$$L_{★}=\max\left\{\prod_{i}\left|B_{i}\right|:⌀\neq B_{i}\subseteq A_{i},\;\prod_{i}B_{i}\subseteq G\right\}.$$

*Each zero-temperature accumulation point is uniform on a maximizing Cartesian subset $\prod_{i}B_{i}\subseteq G$, up to nonuniqueness among subsets with size $L_{★}$, and its entropy is $\log L_{★}$.*

**Proof of Corollary** **2.** Let $p_{0}$ be a zero-temperature accumulation point. In light of Theorem 4,

$$P_{p_{0}}\left(G\right)=1,\;H\left(P_{p_{0}}\right)=\max\left\{H\left(P_{p}\right):P_{p}\left(G\right)=1\right\}.$$

For any product law $P_{p}={⨂}_{i}p_{i}$, let $B_{i}=\mathrm{supp}\left(p_{i}\right)$. Since $P_{p}\left(\sigma \right)=\prod_{i}p_{i}\left(\sigma_{i}\right)$, its support is $\mathrm{supp}\left(P_{p}\right)=\prod_{i}B_{i}$. Thus,

$$P_{p}\left(G\right)=1\;⟺\;\prod_{i}B_{i}\subseteq G.$$

Thus every feasible product law in the entropy maximization of (24) is supported on a Cartesian subset of the ground-state set.

For a given Cartesian support $\prod_{i}B_{i}\subseteq G$, entropy additivity determines

$$H\left(P_{p}\right)=\sum_{i}H\left(p_{i}\right)\leq \sum_{i}\log|B_{i}|=\log\prod_{i}\left|B_{i}\right|,$$

where equality holds if and only if each $p_{i}$ is uniform on $B_{i}$. In that case $P_{p}$ is uniform on $\prod_{i}B_{i}$. Therefore, maximizing entropy over all product laws supported on *G* is equivalent to maximizing the cardinality of a Cartesian subset of *G*:

$$L^{★}=\max\left\{\prod_{i}\left|B_{i}\right|:⌀\neq B_{i}\subseteq A_{i},prod_{i}B_{i}\subseteq G\right\}.$$

Consequently, each zero-temperature accumulation point is uniform on a maximizing Cartesian subset of *G*, and $H\left(P_{p_{0}}\right)=\log L^{★}$. If several distinct Cartesian subsets attain cardinality $L^{★}$, the maximizing product law, and hence the accumulation point, need not be unique.    □

This differs from the exact Gibbs limit, which is uniform over all ground states. If two optimal configurations cannot be mixed coordinatewise without creating a suboptimal configuration, a product law cannot represent their mixture; reverse KL selects one compatible rectangle, often a single mode.

#### 2.3.1. Approximate Optimization, Rounding, and Replicas

Global minimization is not what a generic gradient approach guarantees. The following theorem sheds light on exactly where numerical error enters.

**Theorem** **5** (Approximate solution and extraction guarantees)**.** *Suppose $\hat{p}$ is $\varepsilon_{\mathrm{opt}}$-optimal:*

$$F_{T}\left(\hat{p}\right)\leq \min_{p}F_{T}\left(p\right)+\varepsilon_{\mathrm{opt}},\;a_{T}=T\log M+\varepsilon_{\mathrm{opt}}.$$

*Then*

(26) $$E_{P_{\hat{p}}}\left[E-E_{★}\right]\leq a_{T}.$$

*If $\hat{\sigma }∼P_{\hat{p}}$, then for any r>0,*

(27) $$P\left\{E\left(\hat{\sigma }\right)-E_{★}>r\right\}\leq \min\left\{1,a_{T}/r\right\}.$$

*If ${\hat{\sigma }}^{\left(1\right)},\ldots ,{\hat{\sigma }}^{\left(R\right)}$ are independently sampled from $P_{\hat{p}}$, then*

(28) $$P\left\{\min_{1\leq s\leq R}E\left({\hat{\sigma }}^{\left(s\right)}\right)-E_{★}>r\right\}\leq \min\left\{1,{\left(a_{T}/r\right)}^{R}\right\}.$$

*Finally, deterministic coordinatewise mode rounding satisfies*

(29) $$E\left(\sigma^{\mathrm{mode}}\right)-E_{★}\leq \frac{a_{T}}{\rho_{\mathrm{mode}}}\leq Ma_{T},$$

*where $\sigma_{i}^{\mathrm{mode}}\in {arg max}_{a}{\hat{p}}_{i}\left(a\right)$ and $\rho_{\mathrm{mode}}=P_{\hat{p}}\left(\sigma^{\mathrm{mode}}\right)$.*

**Proof of Theorem** **5.** Since a ground-state point mass is feasible, $\min_{p}F_{T}\left(p\right)\leq E_{★}$. Hence,

$$U\left(\hat{p}\right)-E_{★}=F_{T}\left(\hat{p}\right)+TH\left(P_{\hat{p}}\right)-E_{★}\leq \varepsilon_{\mathrm{opt}}+T\log M=a_{T}.$$

Inequality (27) is obtained by applying Markov’s inequality to the nonnegative random variable $E\left(\hat{\sigma }\right)-E^{★}$, whose expectation is bounded by $a_{T}$ in terms of (26). Since the *R* samples are independent, the probability that all of them have excess energy greater than *r* is at most ${\left(a_{T}/r\right)}^{R}$, which yields (28). For the mode $s=\sigma^{\mathrm{mode}}$,

$$a_{T}\geq E_{P_{\hat{p}}}\left(E-E_{★}\right)\geq P_{\hat{p}}\left(s\right)\left\{E\left(s\right)-E_{★}\right\}.$$

Since $P_{\hat{p}}$ is a product law and $\sigma_{i}^{\mathrm{mode}}\in \arg\max_{a}{\hat{p}}_{i}\left(a\right)$, one gets

$$\rho_{\mathrm{mode}}=P_{\hat{p}}\left(\sigma^{\mathrm{mode}}\right)=\prod_{i}\max_{a}{\hat{p}}_{i}\left(a\right).$$

For each *i*, $\max_{a}{\hat{p}}_{i}\left(a\right)\geq 1/K_{i}$, since the $K_{i}$ probabilities sum to one. Thus, $\rho_{\mathrm{mode}}\geq \prod_{i}K_{i}^{-1}=M^{-1}$. Combining this with $a_{T}\geq \rho_{\mathrm{mode}}\left[E\left(\sigma^{\mathrm{mode}}\right)-E^{★}\right]$ yields (29).    □

The random-sampling bound can be far sharper than the worst-case mode bound. The latter necessarily contains the mode mass: a product expectation can be small even when a low-probability coordinatewise mode has poor energy. In actuality, evaluating both the mode and several samples from each final product law is therefore theoretically defensible.

The *R* batched FEM replicas in [25] are optimized from different initial conditions rather than sampled from a single globally optimized law. If a random initialization reaches a target basin with probability *s*, and replicas behave independently, the best-of-*R* failure probability is ${\left(1-s\right)}^{R}$. This is an optimization-search guarantee, not an increase in the expressive power of the product family; *s* is problem- and optimizer-dependent and has no positive universal lower bound.

#### 2.3.2. Why Low Temperature Creates Many Traps

Call $\sigma^{\circ }$ a strict one-flip local optimum if

(30) $$\gamma_{i}=\min_{a\neq \sigma_{i}^{\circ }}\left\{E\left(a,\sigma_{-i}^{\circ }\right)-E\left(\sigma^{\circ }\right)\right\}>0\;\mathrm{for}\;\mathrm{every}\;i.$$

Let $C_{i}=\sum_{j\neq i}c_{ij}$, with $c_{ij}$ from (16).

**Proposition** **4** (Stationary points near discrete local optima)**.** *Fix r∈(0,1). If*

(31) $$C_{i}r\leq \gamma_{i}/2,\;\left(K_{i}-1\right)e^{-\beta \gamma_{i}/2}\leq r\;\mathrm{for}\;\mathrm{every}\;i,$$

*then the mean-field map $S_{\beta }$ has a fixed point in*

$$B_{r}\left(\sigma^{\circ }\right)=\left\{p:p_{i}\left(\sigma_{i}^{\circ }\right)\geq 1-r\;\mathrm{for}\;\mathrm{every}\;i\right\}.$$

*At that fixed point,*

(32) $$1-p_{i}\left(\sigma_{i}^{\circ }\right)\leq \left(K_{i}-1\right)\exp\left[-\beta \left(\gamma_{i}-\sum_{j\neq i}c_{ij}\left\{1-p_{j}\left(\sigma_{j}^{\circ }\right)\right\}\right)\right].$$

*If its restricted Hessian (20) is positive definite, this point is a strict local minimum.*

**Proof of Proposition** **4.** For $a\neq \sigma_{i}^{\circ }$, define $D_{i}^{a}\left(x_{-i}\right)=E\left(a,x_{-i}\right)-E\left(\sigma_{i}^{\circ },x_{-i}\right)$. For $X_{-i}∼P_{p,-i}$, by changing the coordinates of $X_{-i}$ one at a time to those of $\sigma_{-i}^{\circ }$ and using the definition of $c_{ij}$ in (16), we obtain

$$D_{i}^{a}\left(X_{-i}\right)\geq D_{i}^{a}\left(\sigma_{-i}^{\circ }\right)-\sum_{j\neq i}c_{ij}1\left\{X_{j}\neq \sigma_{j}^{\circ }\right\}.$$

Taking expectations and using $D_{i}^{a}\left(\sigma_{-i}^{\circ }\right)\geq \gamma_{i}$, one obtains

$$E_{P_{p,-i}}D_{i}^{a}\geq \gamma_{i}-\sum_{j\neq i}c_{ij}\left\{1-p_{j}\left(\sigma_{j}^{\circ }\right)\right\}.$$

Since $p\in B_{r}\left(\sigma^{\circ }\right)$, $1-p_{j}\left(\sigma_{j}^{\circ }\right)\leq r$, and hence

$$E_{P_{p,-i}}D_{i}^{a}\geq \gamma_{i}-C_{i}r\geq \gamma_{i}/2.$$

For $a\neq \sigma_{i}^{\circ }$, the softmax equation obtains

$$\frac{{\left(S_{\beta }p\right)}_{i}\left(a\right)}{{\left(S_{\beta }p\right)}_{i}\left(\sigma_{i}^{\circ }\right)}=\exp\;\left\{-\beta \left[e_{i}\left(a;p_{-i}\right)-e_{i}\left(\sigma_{i}^{\circ };p_{-i}\right)\right]\right\}=\exp\;\left\{-\beta E_{P_{p,-i}}D_{i}^{a}\right\}.$$

Since $E_{P_{p,-i}}D_{i}^{a}\geq \gamma_{i}/2$, it follows that

$${\left(S_{\beta }p\right)}_{i}\left(a\right)\leq e^{-\beta \gamma_{i}/2}.$$

Thus,

$$1-{\left(S_{\beta }p\right)}_{i}\left(\sigma_{i}^{\circ }\right)=\sum_{a\neq \sigma_{i}^{\circ }}{\left(S_{\beta }p\right)}_{i}\left(a\right)\leq \left(K_{i}-1\right)e^{-\beta \gamma_{i}/2}\leq r.$$

Hence, for each $p\in B_{r}\left(\sigma^{\circ }\right)$ and each *i*,

$${\left(S_{\beta }p\right)}_{i}\left(\sigma_{i}^{\circ }\right)\geq 1-r,$$

and hence $S_{\beta }\left(B_{r}\left(\sigma^{\circ }\right)\right)\subseteq B_{r}\left(\sigma^{\circ }\right)$. The set $B_{r}\left(\sigma^{\circ }\right)$ is nonempty, compact, and convex, and $S_{\beta }$ is continuous. Brouwer’s fixed-point theorem therefore yields a point $p^{★}\in B_{r}\left(\sigma^{\circ }\right)$ satisfying $S_{\beta }p^{★}=p^{★}$.

At this fixed point, retaining the individual error probabilities rather than replacing them by the uniform bound *r* gets

$$E_{P_{p^{★},-i}}D_{i}^{a}\geq \gamma_{i}-\sum_{j\neq i}c_{ij}\left\{1-p_{j}^{★}\left(\sigma_{j}^{\circ }\right)\right\}.$$

Using the softmax fixed-point equation and summing over $a\neq \sigma_{i}^{\circ }$, we therefore deduce (32). Finally, since a fixed point of $S_{\beta }$ is an interior stationary point of $F_{\beta }$, positive definiteness of the restricted Hessian on the simplex tangent space implies, by the second-order sufficient condition, that $p^{★}$ is a strict local minimum.    □

Therefore, each sufficiently robust one-flip optimum can seed a low-temperature stationary branch. Entropy smooths the landscape at the start of annealing, but it cannot remove the combinatorial multiplicity after the weak-interaction regime ends.

## 3. Annealing, Stability, and Computation

This section investigates several computational implications of the preceding variational analysis. It first examines the tracking behavior of damped mean-field iterations along the temperature-dependent solution path within the contractive regime. The sensitivity of FEM solutions to approximate or noisy energy evaluations is then quantified. Finally, the per-step computational complexity for factorized energies is analyzed, clarifying when exact objective and gradient evaluations remain tractable despite the difficulty of global optimization.

### 3.1. Tracking the High-Temperature Solution Path

The next theorem unveils an idealized damped mean-field update, which is a baseline for annealing analysis.

**Theorem** **6** (Adiabatic tracking under contraction)**.** *Assume $\beta_{t}\in \left[0,\bar{\beta }\right]$ and $\bar{\alpha }=\bar{\beta }C/4<1$. Let $p_{\beta }$ be the unique fixed point and set*

$$B=\underset{p,i}{\sup}{\mathrm{osc}}_{a\in A_{i}}e_{i}\left(a;p_{-i}\right)\leq R_{E},\;K=\frac{B}{4(1-\bar{\alpha })}.$$

*Then,*

(33) $$d_{\infty }\left(p_{\beta },p_{\beta^{\prime }}\right)\leq K\left|\beta -\beta^{\prime }\right|.$$

*For the update*

$$p^{\left(t+1\right)}=\left(1-\eta \right)p^{\left(t\right)}+\eta S_{\beta_{t+1}}p^{\left(t\right)},\;0<\eta \leq 1,$$

*write $q=1-\eta (1-\bar{\alpha })$. If $\delta_{\beta }=\max_{t}\left|\beta_{t+1}-\beta_{t}\right|$, then*

(34) $$d_{\infty }\left(p^{\left(t\right)},p_{\beta_{t}}\right)\leq q^{t}d_{\infty }\left(p^{\left(0\right)},p_{\beta_{0}}\right)+\frac{K\delta_{\beta }}{\eta (1-\bar{\alpha })}.$$

**Proof of Theorem** **6.** Applying Lemma 1 to the same conditional energy vector at two inverse temperatures, one gives

$$d_{\infty }\left(S_{\beta }p,S_{\beta^{\prime }}p\right)\leq B\left|\beta -\beta^{\prime }\right|/4.$$

Combining this with contraction and the fixed-point identities obtains

$$d_{\infty }\left(p_{\beta },p_{\beta^{\prime }}\right)\leq \bar{\alpha }d_{\infty }\left(p_{\beta },p_{\beta^{\prime }}\right)+B\left|\beta -\beta^{\prime }\right|/4,$$

which determines (33).

Define the damped map

$$T_{\beta }\left(p\right)=\left(1-\eta \right)p+\eta S_{\beta }p.$$

By means of convexity of total variation under mixtures and the uniform contraction bound $d_{\infty }\left(S_{\beta }p,S_{\beta }q\right)\leq \bar{\alpha }d_{\infty }\left(p,q\right)$,

$$d_{\infty }\left(T_{\beta }p,T_{\beta }q\right)\leq \left\{\left(1-\eta \right)+\eta \bar{\alpha }\right\}d_{\infty }\left(p,q\right)=q\;d_{\infty }\left(p,q\right),$$

where $q=1-\eta (1-\bar{\alpha })<1$. Moreover, according to $S_{\beta_{t+1}}p_{\beta_{t+1}}=p_{\beta_{t+1}}$, the point $p_{\beta_{t+1}}$ is also a fixed point of $T_{\beta_{t+1}}$. Thus, writing $e_{t}=d_{\infty }\left(p^{\left(t\right)},p_{\beta_{t}}\right)$, one gets

$$\begin{array}{cc} e_{t+1} & =d_{\infty }\;\left(T_{\beta_{t+1}}p^{\left(t\right)},T_{\beta_{t+1}}p_{\beta_{t+1}}\right)\leq q\;d_{\infty }\left(p^{\left(t\right)},p_{\beta_{t+1}}\right)\leq q\left\{e_{t}+d_{\infty }\left(p_{\beta_{t}},p_{\beta_{t+1}}\right)\right\}\\ & \leq q\left\{e_{t}+K\left|\beta_{t+1}-\beta_{t}\right|\right\}\leq qe_{t}+qK\delta_{\beta }, \end{array}$$

where the penultimate inequality follows from (33). Iterating this recursion yields

$$e_{t}\leq q^{t}e_{0}+K\delta_{\beta }\sum_{j=1}^{t}q^{j}=q^{t}e_{0}+\frac{q(1-q^{t})}{1-q}K\delta_{\beta }.$$

Since q≤1 and $1-q=\eta (1-\bar{\alpha })$, one has

$$e_{t}\leq q^{t}e_{0}+\frac{K\delta_{\beta }}{\eta (1-\bar{\alpha })},$$

which is (34).    □

The bound determines a precise meaning to “slow enough” annealing before the first bifurcation. After contractivity is lost, a universal path-tracking guarantee requires stronger, branch-specific curvature assumptions.

### 3.2. Stability to an Approximate or Noisy Energy

**Theorem** **7** (Energy-perturbation stability)**.** *Let E and $\tilde{E}$ satisfy $∥E-\tilde{E}{∥}_{\infty }\leq \varepsilon_{E}$. Their optimal free-energy values differ by at most $\varepsilon_{E}$. If both mean-field maps are contractive with a common factor α<1, their unique solutions satisfy*

(35) $$d_{\infty }\left(p_{\beta },{\tilde{p}}_{\beta }\right)\leq \frac{\beta \varepsilon_{E}}{2(1-\alpha )}.$$

**Proof of Theorem** **7.** For any *p*, the two free-energy objectives have the same entropy term, and therefore

$$F_{\beta ,E}\left(p\right)-F_{\beta ,\tilde{E}}\left(p\right)=U_{E}\left(p\right)-U_{\tilde{E}}\left(p\right)=E_{P_{p}}\left[E\left(\sigma \right)-\tilde{E}\left(\sigma \right)\right].$$

Thus, by virtue of $∥E-\tilde{E}{∥}_{\infty }\leq \varepsilon_{E}$,

$$\left|F_{\beta ,E}\left(p\right)-F_{\beta ,\tilde{E}}\left(p\right)\right|\leq E_{P_{p}}|E\left(\sigma \right)-\tilde{E}\left(\sigma \right)|\leq ∥E-\tilde{E}{∥}_{\infty }\leq \varepsilon_{E}.$$

Since the preceding bound holds uniformly for each *p*, one infers

$$F_{\beta ,E}\left(p\right)\leq F_{\beta ,\tilde{E}}\left(p\right)+\varepsilon_{E}\;\mathrm{for}\;\mathrm{all}\;p.$$

Taking the minimum over *p* on the left and estimating the right-hand side at a minimizer of

$$F_{\beta ,\tilde{E}}$$

gives

$$\min_{p}F_{\beta ,E}\left(p\right)\leq \min_{p}F_{\beta ,\tilde{E}}\left(p\right)+\varepsilon_{E}.$$

Interchanging *E* and $\tilde{E}$ similarly deduces

$$\min_{p}F_{\beta ,\tilde{E}}\left(p\right)\leq \min_{p}F_{\beta ,E}\left(p\right)+\varepsilon_{E}.$$

Combining the two inequalities yields

$$\left|\min_{p}F_{\beta ,E}\left(p\right)-\min_{p}F_{\beta ,\tilde{E}}\left(p\right)\right|\leq \varepsilon_{E},$$

which infers the optimal-value bound.

For each *i*, *a*, and *p*, the corresponding conditional energies satisfy

$$\left|e_{i,E}\left(a;p_{-i}\right)-e_{i,\tilde{E}}\left(a;p_{-i}\right)\right|\leq E_{P_{p,-i}}\left|E\left(a,\sigma_{-i}\right)-\tilde{E}\left(a,\sigma_{-i}\right)\right|\leq \varepsilon_{E}.$$

Thus,

$${\mathrm{osc}}_{a}\left(e_{i,E}\left(a;p_{-i}\right)-e_{i,\tilde{E}}\left(a;p_{-i}\right)\right)\leq 2\varepsilon_{E}.$$

In terms of Lemma 1, one confirms

$$d_{\mathrm{TV}}\left({\left(S_{\beta ,E}p\right)}_{i},{\left(S_{\beta ,\tilde{E}}p\right)}_{i}\right)\leq \frac{\beta \varepsilon_{E}}{2},$$

and taking the maximum over *i* yields

$$d_{\infty }\left(S_{\beta ,E}p,S_{\beta ,\tilde{E}}p\right)\leq \frac{\beta \varepsilon_{E}}{2}.$$

Now use the fixed-point identities $p_{\beta }=S_{\beta ,E}p_{\beta }$, ${\tilde{p}}_{\beta }=S_{\beta ,\tilde{E}}{\tilde{p}}_{\beta }$. Adding and subtracting $S_{\beta ,E}{\tilde{p}}_{\beta }$ and applying the triangle inequality and the common contraction bound with factor α, we obtain

$$\begin{array}{cc} d_{\infty }\left(p_{\beta },{\tilde{p}}_{\beta }\right) & \leq d_{\infty }\left(S_{\beta ,E}p_{\beta },S_{\beta ,E}{\tilde{p}}_{\beta }\right)+d_{\infty }\left(S_{\beta ,E}{\tilde{p}}_{\beta },S_{\beta ,\tilde{E}}{\tilde{p}}_{\beta }\right)\\ & \leq \alpha d_{\infty }\left(p_{\beta },{\tilde{p}}_{\beta }\right)+\frac{\beta \varepsilon_{E}}{2}. \end{array}$$

Since α<1, rearranging derives (35).    □

The factor β is unavoidable: low temperature amplifies small energy errors. This matters when U(p) or its gradient is estimated by stochastic sampling, low precision arithmetic, or a learned surrogate.

### 3.3. Per-Step Complexity for Factorized Energies

Suppose the energy admits a factor-graph representation

(36) $$E\left(\sigma \right)=\sum_{a\in F}E_{a}\left(\sigma_{S_{a}}\right),\;\left|S_{a}\right|\leq k,\;K_{i}\leq K.$$

Then,

(37) $$U\left(p\right)=\sum_{a\in F}\sum_{\sigma_{S_{a}}}E_{a}\left(\sigma_{S_{a}}\right)\prod_{i\in S_{a}}p_{i}\left(\sigma_{i}\right).$$

**Proposition** **5** (Arithmetic cost)**.** *For (36), the exact objective and all factor-to-variable gradient messages can be evaluated in*

(38) $$O\;\left(\sum_{a\in F}\left|S_{a}\right|K^{|S_{a}|}\right)=O(|F|kK^{k})$$

*arithmetic operations, plus O(dK) for entropy. A batch of R replicas multiplies the arithmetic work and parameter memory by R, but the replica dimension is embarrassingly parallel. Hence, bounded arity and sparse factors determine polynomial per-step cost even though global free-energy minimization can remain NP-hard.*

**Proof of Proposition** **5.** For a factor a∈F, set $s_{a}=\left|S_{a}\right|\leq k$ and

$$U_{a}\left(p\right)=\sum_{\sigma_{S_{a}}}E_{a}\left(\sigma_{S_{a}}\right)\prod_{i\in S_{a}}p_{i}\left(\sigma_{i}\right),$$

so that $U\left(p\right)=\sum_{a\in F}U_{a}\left(p\right)$. Since each variable has at most *K* states, the sum defining $U_{a}$ contains at most $K^{s_{a}}$ local configurations. Evaluating the factor value and the corresponding product weight for every local configuration therefore costs $O(K^{s_{a}})$ arithmetic operations up to a factor linear in $s_{a}$.

For each $i\in S_{a}$ and $x\in A_{i}$, the factor-to-variable derivative is

$$\frac{\partial U_{a}}{\partial p_{i}\left(x\right)}=\sum_{\begin{array}{c} \sigma_{S_{a}}:\sigma_{i}=x \end{array}}E_{a}\left(\sigma_{S_{a}}\right)\prod_{j\in S_{a}\setminus \left\{i\right\}}p_{j}\left(\sigma_{j}\right).$$

All such messages can be accumulated in a single enumeration of the $K^{s_{a}}$ local configurations. For each configuration, the products excluding one coordinate can be formed for all $i\in S_{a}$ in $O(s_{a})$ time, for example by prefix–suffix products, and the resulting contribution is added to the message indexed by $\left(i,\sigma_{i}\right)$. Thus the objective contribution and all messages associated with factor *a* require $O(s_{a}K^{s_{a}})$ operations. Summing over factors gives

$$O\;\left(\sum_{a\in F}\left|S_{a}\right|K^{|S_{a}|}\right)\leq O(|F|kK^{k}),$$

which proves (38).

The entropy term decomposes as

$$-H\left(P_{p}\right)=\sum_{i=1}^{d}\sum_{x\in A_{i}}p_{i}\left(x\right)\log p_{i}\left(x\right),$$

so evaluating it and its coordinate derivatives entails $O\left(\sum_{i}K_{i}\right)=O\left(dK\right)$ additional operations. Repeating the same calculations for *R* independent replicas multiplies both arithmetic work and parameter storage by *R*, and this dimension is embarrassingly parallel since no operation couples different replicas. It is noteworthy that Proposition 5 concerns exact per-step evaluation only and does not imply polynomial-time global minimization of the nonconvex free-energy objective.    □

Indeed, a polynomial-time algorithm that globally minimized (2) for arbitrary energies through sufficiently large β would solve any instance of (1), contradicting standard complexity assumptions for NP-hard COPs [2]. The tractability guarantee is therefore for each iteration and, in the contractive regime, for convergence to the unique high-temperature solution. However, it is not a general low-temperature global guarantee.

## 4. Annealed FEM Algorithm and Reproducible Experiments

The foregoing conclusions distinguish three mechanisms that can otherwise be conflated in an implementation: entropy controls the high-temperature geometry, annealing moves the objective through a family of landscapes, and replicas enhance the chance of entering a favorable basin. This section turns that distinction into an explicit algorithm and tests its observable consequences on small instances for which the true optimum can be certified. The experiments are deliberately diagnostic rather than a large-scale solver benchmark.

### 4.1. A Batched Annealed Algorithm

Algorithm 1 implements the exact objective in (2), which applies the centered gradient (7) to *R* product laws in parallel and allows any first-order optimizer. Centering the logits after every update fixes the softmax gauge without changing the marginals. The schedule may be specified stepwise, as presented, or interpolated continuously. The output rule evaluates both marginal modes and optional samples since Theorem 5 yields different guarantees for these two candidate classes.
**Algorithm 1** Annealed free-energy machine with batched replicas**Require:** 
Energy *E* or its factor graph; inverse temperatures $0<\beta_{1}\leq \ldots \leq \beta_{M}$; *R* replicas; $S_{m}$ updates per stage; first-order optimizer Opt; final samples Q≥0  1:Initialize $h_{i,a}^{\left(r\right)}$ near zero for all r,i,a, independently  2:**for** m=1,…,M **do**  3:    **for** $s=1,\ldots ,S_{m}$ **do**  4:        $p_{i}^{\left(r\right)}\leftarrow \mathrm{softmax}\left(h_{i}^{\left(r\right)}\right)$ for every r,i  5:        Evaluate $U(p^{\left(r\right)})$ and $\partial U/\partial p_{i}^{\left(r\right)}\left(a\right)$ factorwise  6:        $g_{i}^{\left(r\right)}\left(a\right)\leftarrow \partial U/\partial p_{i}^{\left(r\right)}\left(a\right)+\beta_{m}^{-1}\left\{\log p_{i}^{\left(r\right)}\left(a\right)+1\right\}$  7:        $G_{i}^{\left(r\right)}\left(a\right)\leftarrow p_{i}^{\left(r\right)}\left(a\right)\left[g_{i}^{\left(r\right)}\left(a\right)-\sum_{b}p_{i}^{\left(r\right)}\left(b\right)g_{i}^{\left(r\right)}\left(b\right)\right]$  8:        h←Opt(h,G)  9:        $h_{i,a}^{\left(r\right)}\leftarrow h_{i,a}^{\left(r\right)}-K_{i}^{-1}\sum_{b}h_{i,b}^{\left(r\right)}$ for every r,i,a10:    **end for**11:**end for**12:Form C from every coordinatewise mode and *Q* independent samples from each final product law13:**return** ${arg min}_{\sigma \in C}E\left(\sigma \right)$

For a sparse factor graph, each update has the cost in Proposition 5; no enumeration of the full configuration space is required. Near-zero logits correspond to near-uniform marginals, which is justified by Theorem 2. The tracking theorem bears gradual changes of β while the contraction condition is established. After that condition fails, Algorithm 1 remains a heuristic: neither the temperature schedule nor the use of an adaptive optimizer supplies a global low-temperature convergence theorem.

### 4.2. Controlled Experimental Design

Three binary problem families are studied with d=16 variables and 20 independent instances per family. Sparse Ising models use edge density 0.35, Gaussian couplings scaled by $d^{-1/2}$, and weak Gaussian fields of standard deviation 0.08. Weighted MaxCut instances use Erdős–Rényi graphs with edge probability 0.30 and independent edge weights uniform on [0.5, 1.5]. Random Max-3SAT instances include 4d three-literal clauses. Exhaustive enumeration of all $2^{16}$ assignments is used only to calculate $E_{★}$ and $E_{\max}$ for evaluation; FEM energies and gradients are evaluated exactly in terms of local factors.

The annealed approach uses R=64 replicas, logits initialized from $N(0,0.02^{2})$, 500 adaptive-gradient updates with learning rate 0.08, and an exponential schedule from β=0.05 to 25. Comparisons are made with the same FEM implementation run for 500 updates at the fixed value β=25 and with 64 single-spin simulated-annealing chains following the same inverse-temperature schedule [10,11]. The latter makes 500 proposals for each chain and is included as a stochastic reference, not as a compute-matched ranking. FEM candidates are the coordinatewise modes of the final marginals. For each instance, exact-ground-state success and the normalized excess are recorded as

(39) $$E_{\mathrm{norm}}=\frac{E_{\mathrm{best}}-E_{★}}{E_{\max}-E_{★}}.$$

All hyperparameters were fixed across the three problem families.

### 4.3. Results and Interpretation

Table 1 uncovers means over the 20 instances in each family. Annealing improves on the fixed low-temperature start for Ising and Max-3SAT in both success and excess energy. MaxCut is different: the fixed run succeeds on all instances, whereas annealing succeeds on 75%. A plausible explanation is provided by the label symmetry discussed in Section 6.2. For Potts MaxCut, the uniform law is stationary, so the entropy-dominated early stages of annealing keep the iterates close to a symmetric state until a symmetry-breaking instability is crossed. With a finite update budget, part of the optimization effort may therefore be spent near the symmetric branch, leaving fewer effective iterations for selecting and refining a favorable low-temperature branch. By contrast, the fixed-β run starts directly in the symmetry-broken low-temperature landscape and avoids this delayed transition. This interpretation suggests that the MaxCut anomaly is more naturally attributed to the interaction among symmetry, annealing schedule, optimizer dynamics, and finite budget than to entropy being intrinsically detrimental to MaxCut. The present experiments do not distinguish these mechanisms uniquely, so this explanation should be regarded as consistent with the theoretical analysis rather than as a causal identification.

**Figure 1:**
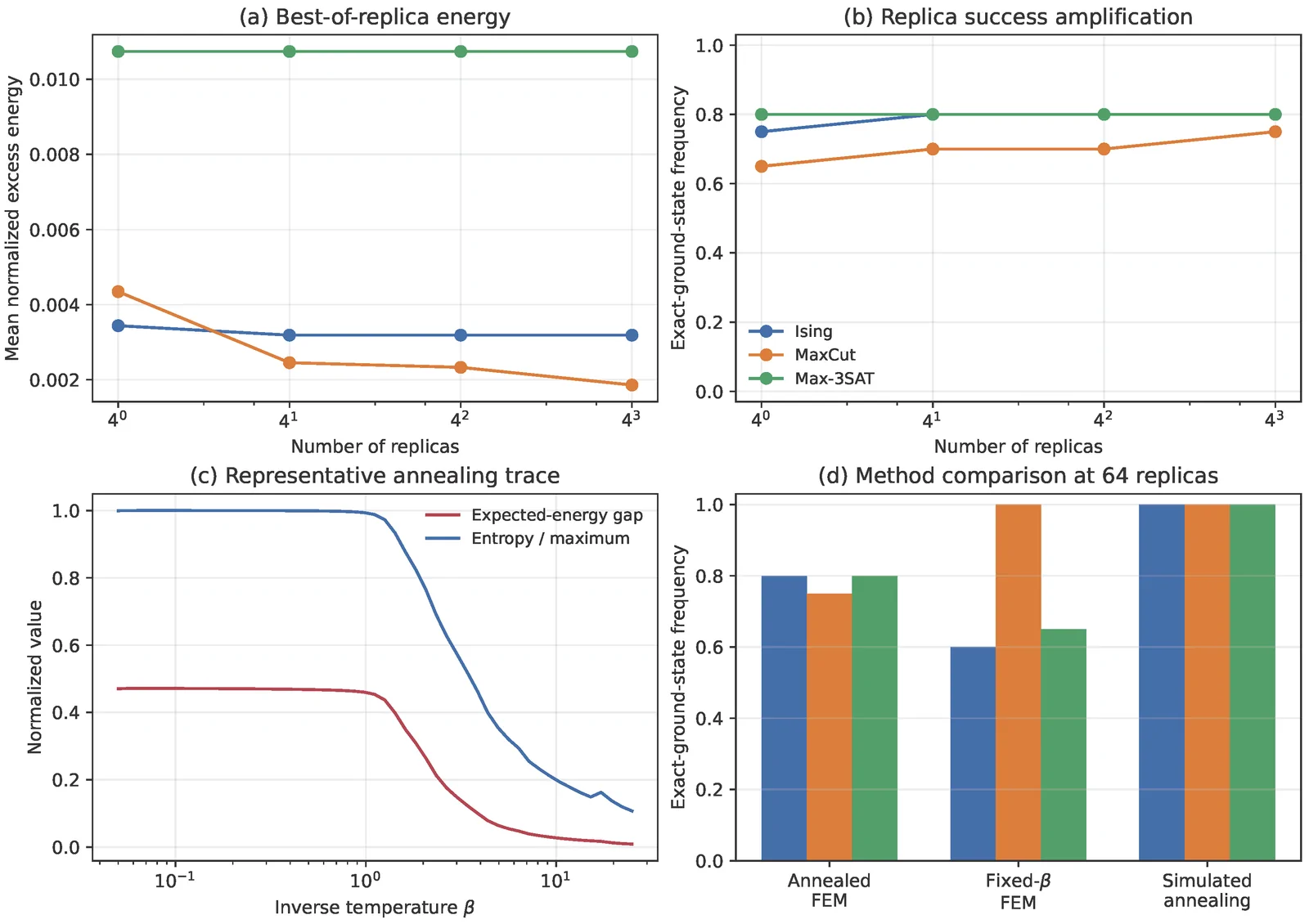
Exactly verifiable synthetic experiments. (**a**) Mean normalized excess and (**b**) exact-ground-state frequency for annealed FEM as the replica count grows. (**c**) Expected-energy gap and entropy along one Ising trajectory. (**d**) Exact success for annealed FEM, fixed-temperature FEM, and simulated annealing with 64 parallel runs.

Figure 1a,b changes the number of annealed FEM replicas while keeping the first R∈{1,4,16,64} runs from the same batch. Replica selection garners a striking improvement for MaxCut, a slight gain for Ising, and no measurable gain for Max-3SAT on these instances. This is consistent with the basin calculation following Theorem 5: replication can amplify a nonzero basin-entry probability but cannot improve the product family or create a favorable basin. Figure 1c presents the predicted thermal behavior on a representative Ising instance: entropy begins near its maximum and falls as the expected-energy gap approaches zero. Figure 1d summarizes the method comparison. Simulated annealing reaches each exact optimum here, but this observation should not be extrapolated to large-scale solver performance since the instances are intentionally small and the workloads are not matched.

## 5. High-Dimensional and Statistical Guarantees

### 5.1. High-Dimensional Phase Diagram

Consider a sequence indexed by the number of variables *d*, with state size $K_{i}\leq K$, energy oscillation $R_{d}$, ground-state gap $\Delta_{d}$, and inverse temperature $\beta_{d}$. The finite-dimensional results immediately infer the scaling laws as follows.

**Corollary** **3** (Three asymptotic regimes)**.** *Let $p_{d}$ be a global FEM minimizer.*

*1*. *If $\beta_{d}R_{d}/d\to 0$, then the average marginal distance to uniform vanishes:*
  $$\frac{1}{d}\sum_{i}d_{\mathrm{TV}}\left(p_{d,i},u_{i}\right)\to 0.$$
  *Joint convergence to uniform follows under the stronger condition $\beta_{d}R_{d}\to 0$.*
*2*. *The expected energy-density error satisfies*
  $$\frac{U\left(p_{d}\right)-E_{★,d}}{d}\leq \frac{\log K}{\beta_{d}}.$$
  *Thus $\beta_{d}\to \infty$ is sufficient for asymptotically optimal energy for each variable.*
*3*. *Exact ground-set concentration can be obtained if*
  $$\frac{\beta_{d}\Delta_{d}}{d\log K}\to \infty .$$

For extensive energies $R_{d}=O\left(d\right)$, the universal bounds separate a high-temperature marginal-uniform regime $\beta_{d}\to 0$ from a low-temperature energy-density regime $\beta_{d}\to \infty$. The intermediate scale $\beta_{d}=\Theta \left(1\right)$ is necessarily model-specific and contains the bifurcations observed in practice. Concentrating on an exact ground state can require a much lower temperature than obtaining a good energy density, especially when $\Delta_{d}$ is small.

Consistent with the qualitative three-stage annealing picture described by Shen et al. [25], the above-mentioned bounds render a quantitative organization of the FEM path into three regimes:
**Regime****Typical scale****Certified or expected behavior**Entropy dominated$\beta_{d}R_{d}/d\to 0$average marginals approach uniform; weak interactions give a unique, contractive fixed pointBifurcation$\beta_{d}=\Theta \left(1\right)$ for extensive energiescurvature and the interaction spectrum determine branch formation; behavior is instance dependentEnergy dominated$\beta_{d}\to \infty$energy density approaches the optimum, while exact ground-set concentration requires the stronger gap-dependent scaling in Corollary 3

### 5.2. Statistical Learning When Energy Is Empirical Risk

The preceding contexts are deterministic and apply to a single COP instance. Random data are required for the genuine statistical-learning interpretation. Suppose $Z_{1},\ldots ,Z_{n}$ are i.i.d., 0≤ℓ(σ;Z)≤1, and

(40) $$E_{n}\left(\sigma \right)=\sum_{t=1}^{n}\ell \left(\sigma ;Z_{t}\right),\;{\hat{R}}_{n}\left(\sigma \right)=n^{-1}E_{n}\left(\sigma \right),\;R\left(\sigma \right)=E\ell \left(\sigma ;Z\right).$$

For a law *Q*, set ${\hat{R}}_{n}\left(Q\right)=E_{Q}{\hat{R}}_{n}\left(\sigma \right)$ and $R\left(Q\right)=E_{Q}R\left(\sigma \right)$. Adding the constant $\beta^{-1}\log M$ and dividing the FEM objective by *n* obtains

(41) $${\hat{R}}_{n}\left(P_{p}\right)+\frac{1}{\eta }D_{\mathrm{KL}}\left(P_{p}∥u\right),$$

where η=βn. Thus, FEM is a product-restricted generalized Gibbs posterior with uniform prior and learning rate η; see [33,34,44].

**Theorem** **8** (PAC-Bayes oracle inequality)**.** *Let δ∈(0,1) and η>0. With probability at least 1−δ over the sample, the exact minimizer ${\hat{P}}_{\eta }\in M$ of (41) satisfies, simultaneously for every comparator Q∈M,*

(42) $$R\left({\hat{P}}_{\eta }\right)\leq R\left(Q\right)+\frac{2\{D_{\mathrm{KL}}\left(Q∥u\right)+\log\left(2/\delta \right)\}}{\eta }+\frac{\eta }{4n}.$$

*If the variational objective in (41) is optimized only to accuracy $\varepsilon_{n}$, add $\varepsilon_{n}$ to the right-hand side. Equivalently, in the original FEM parameterization,*

(43) $$R\left({\hat{P}}_{\beta }\right)\leq R\left(Q\right)+\frac{2\{D_{\mathrm{KL}}\left(Q∥u\right)+\log\left(2/\delta \right)\}}{\beta n}+\frac{\beta }{4}.$$

**Proof of Theorem** **8.** For each given σ, Hoeffding’s lemma implies

$$E\exp\left\{\eta \left(R\left(\sigma \right)-{\hat{R}}_{n}\left(\sigma \right)\right)\right\}\leq e^{\eta^{2}/\left(8n\right)}.$$

Integrating the preceding exponential-moment bound with respect to the uniform prior *u* and exchanging the order of expectation gives

$$E_{D}\;\left[E_{\sigma ∼u}e^{\eta (R\left(\sigma \right)-{\hat{R}}_{n}\left(\sigma \right))}\right]\leq e^{\eta^{2}/\left(8n\right)}.$$

Therefore, by virtue of Markov’s inequality, with probability at least 1−δ/2 over the sample,

$$E_{\sigma ∼u}e^{\eta (R\left(\sigma \right)-{\hat{R}}_{n}\left(\sigma \right))}\leq \frac{2}{\delta }e^{\eta^{2}/\left(8n\right)},$$

or equivalently,

$$\log E_{\sigma ∼u}e^{\eta (R\left(\sigma \right)-{\hat{R}}_{n}\left(\sigma \right))}\leq \log\left(2/\delta \right)+\frac{\eta^{2}}{8n}.$$

On this event, the Donsker–Varadhan change-of-measure inequality [49], applied with

$$f\left(\sigma \right)=\eta (R\left(\sigma \right)-{\hat{R}}_{n}\left(\sigma \right)),$$

implies simultaneously for each law *Q* that

$$\begin{array}{cc} \eta \{R\left(Q\right)-{\hat{R}}_{n}\left(Q\right)\} & \leq D_{\mathrm{KL}}\left(Q∥u\right)+\log E_{u}e^{\eta (R-{\hat{R}}_{n})}\\ & \leq D_{\mathrm{KL}}\left(Q∥u\right)+\log\left(2/\delta \right)+\frac{\eta^{2}}{8n}. \end{array}$$

Dividing by η yields

$$R\left(Q\right)\leq {\hat{R}}_{n}\left(Q\right)+\frac{D_{\mathrm{KL}}\left(Q∥u\right)+\log\left(2/\delta \right)}{\eta }+\frac{\eta }{8n}.$$

Applying the same argument to the reverse deviation ${\hat{R}}_{n}\left(\sigma \right)-R\left(\sigma \right)$, we obtain, by Hoeffding’s lemma, for each given σ,

$$E_{D}\exp\left\{\eta \left({\hat{R}}_{n}\left(\sigma \right)-R\left(\sigma \right)\right)\right\}\leq e^{\eta^{2}/\left(8n\right)}.$$

Integrating with respect to *u* and applying Markov’s inequality shows that, with probability at least 1−δ/2,

$$\log E_{\sigma ∼u}e^{\eta ({\hat{R}}_{n}\left(\sigma \right)-R\left(\sigma \right))}\leq \log\left(2/\delta \right)+\frac{\eta^{2}}{8n}.$$

On this event, the Donsker–Varadhan inequality implies simultaneously for every law *Q* that

$${\hat{R}}_{n}\left(Q\right)\leq R\left(Q\right)+\frac{D_{\mathrm{KL}}\left(Q∥u\right)+\log\left(2/\delta \right)}{\eta }+\frac{\eta }{8n}.$$

Let $E_{+}$ and $E_{-}$ be the events on which the forward and reverse bounds hold, respectively. Since each has probability at least 1−δ/2, the union bound gives

$$\mathrm{Pr}(E_{+}\cap E_{-})\geq 1-\delta .$$

Hence, on a single event $E=E_{+}\cap E_{-}$ of probability at least 1−δ, both inequalities hold simultaneously for all laws *Q*.

For such an event E, since the two bounds hold simultaneously for all laws, the forward bound may in particular be applied to the data-dependent minimizer ${\hat{P}}_{\eta }$. Thus,

$$\begin{array}{cc} R({\hat{P}}_{\eta }) & \leq {\hat{R}}_{n}\left({\hat{P}}_{\eta }\right)+\frac{D_{\mathrm{KL}}\left({\hat{P}}_{\eta }∥u\right)+\log\left(2/\delta \right)}{\eta }+\frac{\eta }{8n}\\ \; & \leq {\hat{R}}_{n}\left(Q\right)+\frac{D_{\mathrm{KL}}\left(Q∥u\right)+\log\left(2/\delta \right)}{\eta }+\frac{\eta }{8n}\\ \; & \leq R\left(Q\right)+\frac{2\{D_{\mathrm{KL}}\left(Q∥u\right)+\log\left(2/\delta \right)\}}{\eta }+\frac{\eta }{4n}. \end{array}$$

If the normalized variational objective in (41) is optimized only to additive accuracy $\varepsilon_{n}$, let ${\tilde{P}}_{\eta }$ be the resulting approximate minimizer. Then, for each Q∈M,

$${\hat{R}}_{n}\left({\tilde{P}}_{\eta }\right)+\frac{1}{\eta }D_{\mathrm{KL}}\left({\tilde{P}}_{\eta }∥u\right)\leq {\hat{R}}_{n}\left(Q\right)+\frac{1}{\eta }D_{\mathrm{KL}}\left(Q∥u\right)+\varepsilon_{n}.$$

Thus, the variational-optimality step above acquires an additive $\varepsilon_{n}$, and the same argument yields

$$R\left({\tilde{P}}_{\eta }\right)\leq R\left(Q\right)+\frac{2\{D_{\mathrm{KL}}\left(Q∥u\right)+\log\left(2/\delta \right)\}}{\eta }+\frac{\eta }{4n}+\varepsilon_{n}.$$

Finally, in light of the relation η=βn from (41), one deduces

$$\frac{2\{D_{\mathrm{KL}}\left(Q∥u\right)+\log\left(2/\delta \right)\}}{\eta }=\frac{2\{D_{\mathrm{KL}}\left(Q∥u\right)+\log\left(2/\delta \right)\}}{\beta n},$$

where $\frac{\eta }{4n}=\frac{\beta }{4}$. Hence, Equation (43) is obtained for exact variational minimization, and an additional $+\varepsilon_{n}$ appears on the right-hand side for an $\varepsilon_{n}$-approximate minimizer of (41). □

Taking $Q=\delta_{\sigma }$ yields $D_{\mathrm{KL}}\left(Q∥u\right)=\log M$. Optimizing the bound at

(44) $$\eta_{★}=\sqrt{8n\{\log M+\log(2/\delta )\}},\;\beta_{★}=\frac{\eta_{★}}{n}$$

yields

(45) $$R\left({\hat{P}}_{\eta_{★}}\right)\leq \min_{\sigma \in A}R\left(\sigma \right)+\sqrt{\frac{2\{\log M+\log(2/\delta )\}}{n}}.$$

The conclusion differs from deterministic annealing. Sending β to infinity is appropriate for minimizing the observed instance energy, whereas the generalization bound favors a finite, sample-size-dependent temperature. If $\log M=\sum_{i}\log K_{i}=O\left(d\right)$, nonvacuous uniform learning requires the data size to dominate the effective combinatorial dimension. This section does not apply when the COP energy has no data-generating interpretation.

## 6. Representative Models and Practical Guidance

This section specializes the general FEM theory to several celebrated combinatorial optimization frameworks and summarizes its practical implications. The framework is first instantiated for QUBO/Ising energies, Potts MaxCut, and MaxSAT, elucidating how the general fixed-point, stability, and complexity results take concrete forms in these problems. The resulting implications for initialization, annealing, replica use, and solution extraction are then discussed, together with the main limitations of the theoretical guarantees.

### 6.1. QUBO and Ising Energies

For spins $\sigma_{i}\in \left\{-1,+1\right\}$, consider

(46) $$E\left(\sigma \right)=-\frac{1}{2}\sigma^{⊤}W\sigma -b^{⊤}\sigma ,\;W=W^{⊤},\;W_{ii}=0.$$

By setting $m_{i}=E\sigma_{i}$, the product free energy becomes

(47) $$F_{\beta }\left(m\right)=-\frac{1}{2}m^{⊤}Wm-b^{⊤}m+\frac{1}{\beta }\sum_{i}\left[\frac{1+m_{i}}{2}\log\frac{1+m_{i}}{2}+\frac{1-m_{i}}{2}\log\frac{1-m_{i}}{2}\right],$$

and (6) reduces to

(48) $$m_{i}=\tanh\left(\beta \left[b_{i}+\sum_{j}W_{ij}m_{j}\right]\right).$$

At b=0, m=0 is stationary, and its Hessian is stated by

(49) $$\nabla^{2}F_{\beta }\left(0\right)=\beta^{-1}I-W.$$

If $\lambda_{\max}\left(W\right)>0$, the first local instability occurs at $\beta_{c}=1/\lambda_{\max}\left(W\right)$. The more conservative global contraction condition is $\beta \max_{i}\sum_{j}\left|W_{ij}\right|<1$. This example distinguishes the spectral local threshold from a row-sum condition certifying global uniqueness.

### 6.2. Potts MaxCut

For *K* labels and

$$E\left(\sigma \right)=-\sum_{(i,j)\in E}W_{ij}\left\{1-1\left(\sigma_{i}=\sigma_{j}\right)\right\},$$

the mean energy is formulated by

(50) $$U\left(p\right)=-\sum_{(i,j)\in E}W_{ij}\left(1-\sum_{a=1}^{K}p_{i}\left(a\right)p_{j}\left(a\right)\right).$$

Up to an action-independent constant, the conditional energy is presented as

$$e_{i}\left(a;p_{-i}\right)=\sum_{j:(i,j)\in E}W_{ij}p_{j}\left(a\right),$$

and hence

(51) $$p_{i}\left(a\right)\propto \exp\left\{-\beta \sum_{j:(i,j)\in E}W_{ij}p_{j}\left(a\right)\right\}.$$

The uniform law is stationary by label symmetry, and its loss of stability is determined jointly by the graph spectrum and the (K−1)-dimensional label tangent space. Balance penalties introduce dense interactions which enlarge the influence constant *C*, thus shrinking the certified high-temperature regime. This symmetry also has algorithmic consequences for annealing. If an annealing schedule allocates too many updates to the stable or weakly unstable symmetric regime, a finite-budget optimizer may have insufficient time after the bifurcation to amplify symmetry-breaking perturbations and settle into a favorable branch. This inspires one possible explanation for the lower annealed-FEM success observed for MaxCut in Table 1 compared with the fixed low-temperature initialization.

### 6.3. MaxSAT

For the unsatisfied-clause energy employed in [25],

(52) $$E\left(\sigma \right)=\sum_{m=1}^{L}\prod_{i\in \partial m}\left\{1-1\left(\sigma_{i}=w_{mi}\right)\right\},$$

the exact product expectation is expressed by

(53) $$U\left(p\right)=\sum_{m=1}^{L}\prod_{i\in \partial m}\left\{1-p_{i}\left(w_{mi}\right)\right\}.$$

For clauses of width at most *k*, Proposition 5 offers $O(Lk2^{k})$ exact arithmetic for each replica and step. The local-optimum result, Proposition 4, is especially relevant because changing one Boolean variable affects only its incident clauses, so the margins and influences can be calculated locally.

### 6.4. Practical Consequences and Limitations

The theories concluded in the above-mentioned sections suggest the following disciplined interpretation of FEM.

1. Initialize uniformly because it is the exact β=0 optimizer. At positive $\beta_{\min}$, Theorem 2 can be employed to determine whether the initialization is genuinely close to the variational optimum.
2. Before the estimated critical region, an influence bound or an empirical Hessian can diagnose whether a unique trackable branch is plausible. In this region, slow annealing has the quantitative meaning in Theorem 6.
3. Beyond the first instability, replicas explore different basins but do not change the mean-field family. Report the best energy together with the number of replicas and initializations, but do not interpret the batch as a correlated variational distribution.
4. Evaluate coordinatewise modes and random samples at termination, and the two extraction approaches have different guarantees in Theorem 5.
5. If a certified lower bound $L\leq E_{★}$ is available from a relaxation, any returned law has the computable certificate
  $$U\left(\hat{p}\right)-E_{★}\leq F_{T}\left(\hat{p}\right)-L+T\log M,$$
  without requiring the global FEM optimum.

Several limitations are noteworthy, on the contrary. First, as Corollary 2 explicitly portrayed, reverse-KL mean field cannot represent correlated mixtures. Second, the global low-temperature rates do not mean that a specific optimizer admits the global variational solution. Third, the Dobrushin bound is conservative and may fail well before an empirical phase transition. Fourth, the PAC-Bayes analysis assumes bounded i.i.d. losses; dependent data, heavy-tailed losses, or a deterministic COP require different tools. Finally, the gradient manipulations used in implementations can depart from the exact gradient (7), and such modifications should be analyzed as a separate algorithm rather than attributed automatically to the free-energy objective.

## 7. Discussion

The central benefit of entropy in FEM is mathematically transparent. At high temperature it selects a unique, nearly uniform and often contractive solution, allowing broad exploration. Lowering the temperature decreases the energy of a globally optimized solution, but also weakens convexification, creates branches near discrete local optima, and magnifies numerical energy errors. At zero temperature the global variational relaxation is exact in objective value because the product family contains each point mass, yet the route to that endpoint remains computationally hard.

The three thermal regimes analyzed here should not be interpreted as a newly proposed phenomenological picture of FEM annealing. Shen et al. [25] already described, through the evolution of entropy, internal energy, and marginal probabilities, a qualitative three-stage process in which entropy favors nearly uniform marginals at high temperature, a distributional transition occurs near a critical inverse temperature, and energy dominates as the temperature approaches zero. The present contribution provides a quantitative characterization of this pipeline for the finite-state product free-energy objective. Specifically, Theorem 2 bounds the high-temperature deviation from uniformity in relative entropy and total variation and identifies the first-order departure from the uniform law; Theorem 3 and Proposition 2 unveil sufficient uniqueness/contraction conditions and an instance-dependent Hessian criterion for the first possible loss of stability; and Theorem 4 displays the explicit low-temperature excess-energy bound $U\left(p_{\beta }\right)-E_{★}\leq \log\left|A\right|/\beta$, together with ground-state concentration and entropy selection under degeneracy. Corollary 3 then organizes these finite-dimensional conclusions into asymptotic entropy-dominated, model-specific bifurcation, and energy-dominated regimes. Thus, three-regime analysis in this work is intended as a rigorous quantitative refinement of the qualitative annealing mechanism proposed in [25], rather than as the first observation of that mechanism.

Several research directions are listed from the analysis. The influence bound could be sharpened by weighted norms or nonbacktracking operators, producing less conservative uniqueness regions for sparse graphs. Below the first bifurcation, branch-specific curvature and barrier estimates may infer invaluable finite-rate annealing guarantees. Structured variational families, TAP or Bethe corrections, and learned autoregressive laws could relieve the rectangular-support limitation while preserving part of FEM’s parallelism [23,50,51]. Moreover, a theory that combines data-dependent generalization, instance perturbations, and nonconvex optimizer dynamics would more closely match large-scale applications. Accordingly, the main novelty is not the individual classical tools, but the unified FEM-specific analysis that separates entropy effects, annealing behavior, numerical optimization, replica selection, and statistical regularization within a single finite-state framework. Finally, a deep extension would replace the uniform reference law by a dynamically updated $Q_{t}$, yielding $U\left(p\right)+\beta^{-1}D_{\mathrm{KL}}\left(P_{p}∥Q_{t}\right)$, which encodes information accumulated during optimization and connects FEM more directly to adaptive biased ensembles, but its convergence and stability require an exclusive analysis because the objective itself becomes history dependent.

## Figures and Tables

**Table 1:** Controlled d=16 experiments. Each entry aggregates 20 instances and 64 replicas or chains. Excess is the mean of (39); uncertainty is one standard error.

Problem	Method	Exact Success	Normalized Excess
Ising	Annealed FEM	0.80	0.00319±0.00175
	Fixed-β FEM	0.60	0.02289±0.00986
	Simulated annealing	1.00	0.00000±0.00000
MaxCut	Annealed FEM	0.75	0.00186±0.00092
	Fixed-β FEM	1.00	0.00000±0.00000
	Simulated annealing	1.00	0.00000±0.00000
Max-3SAT	Annealed FEM	0.80	0.01074±0.00499
	Fixed-β FEM	0.65	0.01784±0.00566
	Simulated annealing	1.00	0.00000±0.00000

## Data Availability

The code and data supporting the results of this article are publicly available at https://github.com/gaowei810213/5 (accessed on 3 September 2026).
